# Life History Traits Reflect Changes in Mediterranean Butterfly Communities Due to Forest Encroachment

**DOI:** 10.1371/journal.pone.0152026

**Published:** 2016-03-21

**Authors:** Jana Slancarova, Alena Bartonova, Michal Zapletal, Milan Kotilinek, Zdenek Faltynek Fric, Nikola Micevski, Vasiliki Kati, Martin Konvicka

**Affiliations:** 1 Faculty of Science, University of South Bohemia, Ceske Budejovice, Czech Republic; 2 Institute of Entomology, Biology Centre CAS, Ceske Budejovice, Czech Republic; 3 Macedonian Entomological Society (ENTOMAK), Skopje, Republic of Macedonia (FYROM); 4 Department of Environmental and Natural Resources Management, University of Patras, Agrinio, Greece; Trier University, GERMANY

## Abstract

The biodiversity of the Southern Balkans, part of the Mediterranean global biodiversity hot-spot, is threatened by land use intensification and abandonment, the latter causing forest encroachment of formerly open habitats. We investigated the impact of forest encroachment on butterfly species richness, community species composition and the representation of life history traits by repeated seasonal visits of 150 one-hectare sites in five separate regions in three countries—Greece, Bulgaria, and the Republic of Macedonia (FYROM—the Former Yugoslav Republic of Macedonia)— 10 replicates for each habitat type of grasslands, open formations and scrub forest within each region. Grasslands and open formations sites hosted in average more species and more red-listed species than scrub forest, while no pattern was found for numbers of Mediterranean species. As shown by ordination analyses, each of the three habitat types hosted distinct butterfly communities, with Mediterranean species inclining either towards grasslands or open formations. Analysing the representation of life history traits revealed that successional development from grasslands and open formations towards scrub forest shifts the community composition towards species overwintering in earlier stages, having fewer generations per year, and inhabiting large European or Eurosiberian (e.g. northern) ranges; it decreases the representation of Mediterranean endemics. The loss of grasslands and semi-open formations due to forest encroachment thus threatens exactly the species that should be the focus of conservation attention in the Mediterranean region, and innovative conservation actions to prevent ongoing forest encroachment are badly needed.

## Introduction

The Mediterranean region of Europe is a global biodiversity hot-spot, due to its exceptional endemism rate, species richness and threat degree [1]. The high diversity of mountain ranges, gorges, peninsulas and islands creates more complex climatic patterns than anywhere in Europe [2]. The great diversity of Mediterranean ecosystems has been further augmented by human activities. As a cradle of human civilisation, the region had been affected, in chronological order, by large herbivores extirpation, forest clearance, pasture, farming, and urbanisation [3]. Although the character of its natural vegetation is still disputed, the high representation of endemics depending on non-wooded conditions suggests that in a pristine state, sizeable parts of the region would be covered by open habitats, maintained by rainstorms, fires, and herbivore actions [4]. In the course of history, traditional agro-pastoral land uses replaced these natural disturbances, maintaining or even enhancing landscape and habitat diversity.

Agricultural intensification in fertile lowlands [5] along with land abandonment and subsequent forest encroachment in less fertile remote areas [6, 7] are recognised as major threats for European biodiversity [8]. Forest encroachment represents a particular risk in the Mediterranean, given the high regional endemism associated with open habitats [9]. The whole situation is complicated by rapid changes in farming patterns in some regions, to which the EU conservation policies do not always respond appropriately [10]. Financial incentives for afforestation too often reflect the “forested Mediterranean” paradigm (cf. [4]). The scale of the problem differs among individual countries. For instance, traditional land use began to decline in Greece with economic growth in the 1970s (e.g., 43% decline in rough grazing: [11]), whereas in Bulgaria, farming has declined following the fall of communism in early 1990s, and in the Republic of Macedonia, traditional farming still persists.

The impact of forest encroachment on Mediterranean invertebrate diversity is poorly studied. Forest encroachment is expected to have a negative effect on invertebrate taxa that prefer open habitats, e.g, due to particular host plant associations, nectar requirements, or temperature requirements [12–14]. For butterflies in particular, a large-scale study from the Iberian Peninsula [15] demonstrated the negative effect of marginal land abandonment, and subsequent forest increase—but no such studies exist from the Apennine and Balkan peninsulas, although data from a Greek nature reserve point to the same pattern [16]. Land abandonment initially increases the species richness, as open woodland species supplement grasslands [17]. This is followed by a loss of sensitive species, which are replaced by generalists (butterflies in Romania and Spain: [18, 19]). Eventually, as new dense forests prevail, only a few closed forest species persist.

Species life history traits have recently gained attention as an alternative path to detect changes of biological communities from a functional point of view, at finite and large scales, besides the classical species turnover approaches [20–22]. In butterflies, traits such as a low number of generations, low mobility and narrow trophic range collectively define specialism, associated with small ranges and a tendency to decline in altered landscapes (e.g. [23]). Another major gradient, defined by the association between larval host plant growth form, butterfly body size and yearly generation numbers, defines the tendency to occur in wooded versus non-wooded habitats [24].

This paper examines the impact of the forest encroachment process that usually follows agro-pastoral land abandonment on butterfly communities on a regional scale, considering three countries in the Southern Balkans: namely Bulgaria, Greece, and the (Former Yugoslav) Republic of Macedonia (hereafter Macedonia). We explore the impact of forest encroachment on (a) butterfly diversity patterns, (b) butterfly community composition, and (c) butterfly life history traits. We hence investigate the ecological and functional response of butterfly communities to land abandonment/forest encroachment in an integrated way, in order to provide specific proposals for their conservation.

## Materials and Methods

### Ethics statement

The study was carried out in accordance with the national laws and permits obtained from authorised institutions: Bulgaria (National Museum of Natural History, Sofia), Greece (Eλληνική Δημοκρατία, Yπoυργείo Περιβάλλοντος, Eνέργειας & Kλιματικής Aλλαγής, Eιδική Γραμματεία Δασών, Γενική Διεύθυνση Aνάπτυξης & Πρoστασίας Δασών & Φυσικών Πόρων, Δ/νση Aισθ. Δασών, Δρυμών και Θήρας Τμήμα Γ & B, No. 170916/1344), and Macedonia (Bird Study and Protection Society of Macedonia). The fieldwork was not carried out in any privately owned nor protected areas. All the butterflies were carefully handled and released after identification; we collected up to five individuals per visit only for taxa not identifiable in the field, for genital preparation and species identification in the lab (not applicable regarding protected species).

### Site selection

**Fig 1:**
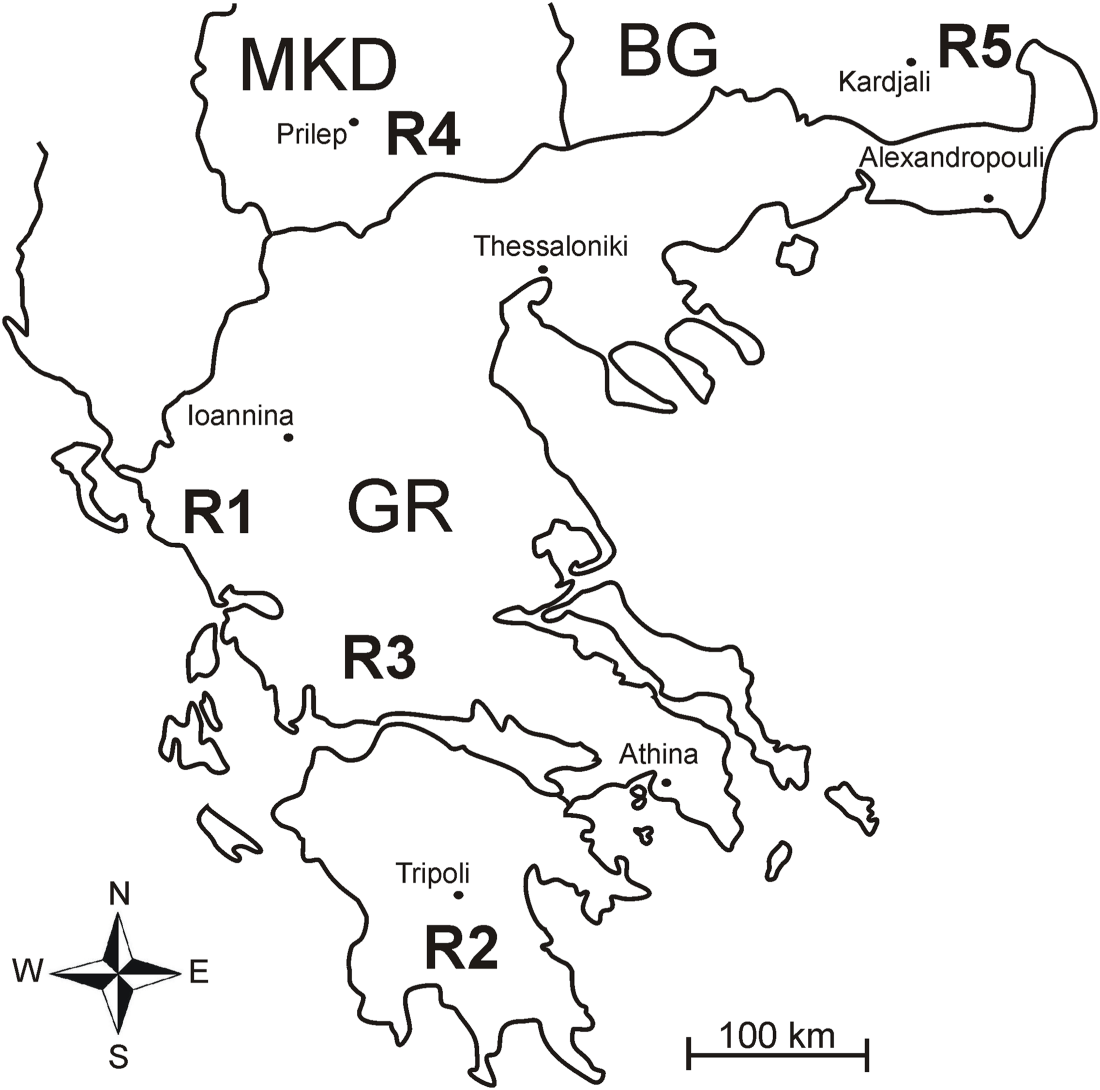
A map of the Southern Balkans showing the five study regions where impacts of Forest encroachment on butterflies were studied. R1 –foothills of Paramythia Mts, NW Greece (Epirus province); R2 –foothills of Taygetos Mts., S Greece (Lakonia); R3 –southern foothills of Giona Mts, Greece (Sterea Ellada); R4 –Macedonia, Prilep environs; R5 –SE Bulgaria (Kardzhali environs). Average aerial distance among regions was 220 km (range 143–320 km).

Our study area was located in the Southern Balkans, encompassing five regions (R1–R5): three in Greece, one in Macedonia, and one in Bulgaria (Fig 1), (S1 Text). We predefined three forest encroachment categories, in terms of woody vegetation cover (> 1.5m), with the help of post-2010 aerial photographs: (a) *Grasslands*–herbaceous vegetation dominance and a woody plant cover less than 5% with tracks of active grazing, (b) *Open formations*–near-even representation of woody and herbaceous cover, (c) *Scrub forest*, with dominance of woody plant vegetation above 70%. We located 30 sampling sites of 1ha standard area in each region (altitude from 10 to 1100 m a. s. l. (mean 440 ± 254 SE)), so as to equally represent the three forest encroachment categories, resulting in an overall number of 150 sites sampled.

### Butterfly sampling

We sampled butterflies during four visits in early spring (April/May 2012), late spring (May/June 2012), summer (July 2013), and late summer (August/September 2012), to well cover butterfly phenology [25]. We recorded butterflies in terms of timed surveys [26] lasting 30 person-minutes per site between 9:00–17:00, under suitable weather, using semi-quantitative abundance categories (1, ≤ 5, ≤10, ≤20, ≤50, ≤100, >100 individuals). For taxa not identifiable in the field, we collected up to five individuals per visit for genital preparation and species identification in the lab. Butterfly nomenclature follows Fauna Europaea [27], Red List categorization according to Van Swaay et al. [28] (S1 Table).

### Life history traits

We considered 15 life history traits for butterflies readily available in literature and reflecting (a) specialism *vs*. generalism (*Feeding index*, *Flight period*, *Generation numbers*, *Migration*, *Overwintering stage*, *Wingspan*), (b) larval feeding habits (*Gregariousness*, *Host plant form*, *Larval feeding mode*, *Myrmecophily*, *Ovum placement*), as well as (c) distribution profile (*Altitudinal range*, *Mountain distribution*, *Range size*, *Range type*). These functional traits are linked with the resilience of the species to environmental or land use change and hence its inherent vulnerability tendency (S2 Table). Information on most of the traits is directly available in literature, or, as in the case of *Feeding index*, easily calculable from published data (see S3 Table). An exception was information on range size, where we used simple numeric coding based on published distribution maps (see S3 Table for references).

### Environmental parameters

We collected the following 15 environmental parameters for each site sampled. Two variables were collected to describe the forest encroachment gradient and were inserted in the model as predictors, namely: *Forest encroachment* was categorically variable with tree levels (*Grassland*, *Open formations*, *Scrub forest*); *Canopy* cover (percentage cover of woody species >1.5m) was a continuous predictor.

Another set of twelve variables was used as covariables in the model to describe (a) the geographical position (*Latitude* (mean: 22.54 ± 1.70), *Longitude* (39.60±1.78)), (b) the topography (*Altitude* (*m*) (441.89±254.07)), *Slope* (three categories: 1: flat (<15%), 2: sloping (<30%), 3: steep (>30%)) and *Exposure* (ranked: SW, S– 5; SE, W– 4; flat– 3; NW, E– 2; N, NE– 1)), (c) the site humidity (*Water* presence (binary value)), (d) human presence and grazing intensity (*Road* presence (binary value), nearest *Village* distance (m) (1610±1014), nearest *Herdsman’s hut* distance (m) (825±631)), as well as (e) *Vegetation composition* (four variables: *Veg1–Veg 4*, which were obtained by recording all vascular plants species with their relative covers (1–3 scale) at each site for a standard time of 60 min during May–June 2013 [29], subjecting thus recorded data to principal component analysis (PCA) and extracting values of four PCA axes (details: S1 Fig)).

For each site visit, we recorded further parameters describing momentary weather conditions, namely *Air temperature*, *Cloudiness* (1: clear to 3: half-sunny), *Wind* (1: calm to 3: moderate breeze), as well as momentary *Nectar* supply, using a simple ranked scale (1: none or a few isolated flower heads, 2: isolated flowering patches, 3: whole site in bloom).

### Data analysis

We transformed the recorded semi-quantitative butterfly abundances to mean numbers of individuals within the respective quantitative intervals, summed this across the four visits, and log-transformed. *Air temperature*, *Cloudiness*, *Wind* and *Nectar* were also summed across the four visits to obtain more detailed scales.

We applied generalised linear models in R [30] (Poisson distribution of the response) to analyze *Forest encroachment* and *Canopy* effects on species richness, numbers of Red-listed species, and numbers of Mediterranean species. For all three response variables, we first tested independent effects of the two primary predictors, considering also polynomial response for *Canopy*. Next, we tested independent effects of all site parameters and visit parameters (i.e., potential nuisance covariables), and used stepwise selection based on all potential covariables, evaluating alternative models’ fits according to the Akaike information criterion (*AIC*) to obtain *covariate models*, defined as models best explaining the response variables without referring to the predictor(s) of interest. Finally, we manually forced the predictors *Forest encroachment* and *Canopy* onto the *covariate models*, thus assessing their effects while statistically controlling for variation due to nuisance variables.

To study changes effects on species composition, we used redundancy analysis (RDA), a constrained linear ordination, using CANOCO 5 [31]. We first computed single-term ordinations for both predictors of interest and all covariables. Next, we defined a *covariate model*, based on forward selection from potential covariables. Finally, we computed partial RDAs with predictors *Forest encroachment* and *Canopy*, controlled for effects of *covariate model* terms. We log (x + 1) transformed and centred species abundances in all RDA analyses, and evaluated significances of the ordinations using the Monte Carlo test (999 permutations).

We used the partial RDAs to analyse the life history traits responses. Because life histories co-vary with phylogeny (e.g. [32]), we constructed a phylogenetic tree of all recorded species, based on published phylogenies, supplemented by formal classification into genera and subgenera (S2 Text). We turned this tree into a patristic distance matrix, representing the distance of any pair of taxa measured along the branches of the phylogenetic tree. We transformed this distance matrix into a set of descriptors using principal coordinate analysis (PCoA), with PCoA scores centred and standardised. Not all PCoA scores are related to response variable, therefore we used their subset selected by forward selection—only descriptors with *p* < 0.04 were included. Finally, we interpreted the species traits responses to *Forest encroachment* and *Canopy* individually for each trait, after removing the variation explained by phylogenetic descriptors. We evaluated each step using the Monte Carlo test (999 permutations).

Raw dataset for all analyses is available as supplementary material S1 Raw Data.

## Results

### Species richness patterns

We recorded 128 species in total (R1: 81, R2: 72, R3: 69, R4: 98, R5: 77), including 11 species from the European Red List (R1: 5, R2: 6, R3: 3, R4: 8, R5: 7) (S1 Table). The mean species richness values per site and region were: R1, 23 (SD 7.6, range 12–37); R2, 15 (6.0, 6–30); R3, 17 (4.8, 6–25); R4, 27 (7.6, 11–42); R5, 24 (4.4, 14–32). The regions R4 and R5 hosted significantly higher per site species richness (Kruskal-Wallis χ^2^ = 50.7, df = 4, P < 0.0001); whereas the region R2 hosted significantly more endangered red-listed species per site (χ^2^ = 18.7, df = 4, P < 0.001).

**Fig 2:**
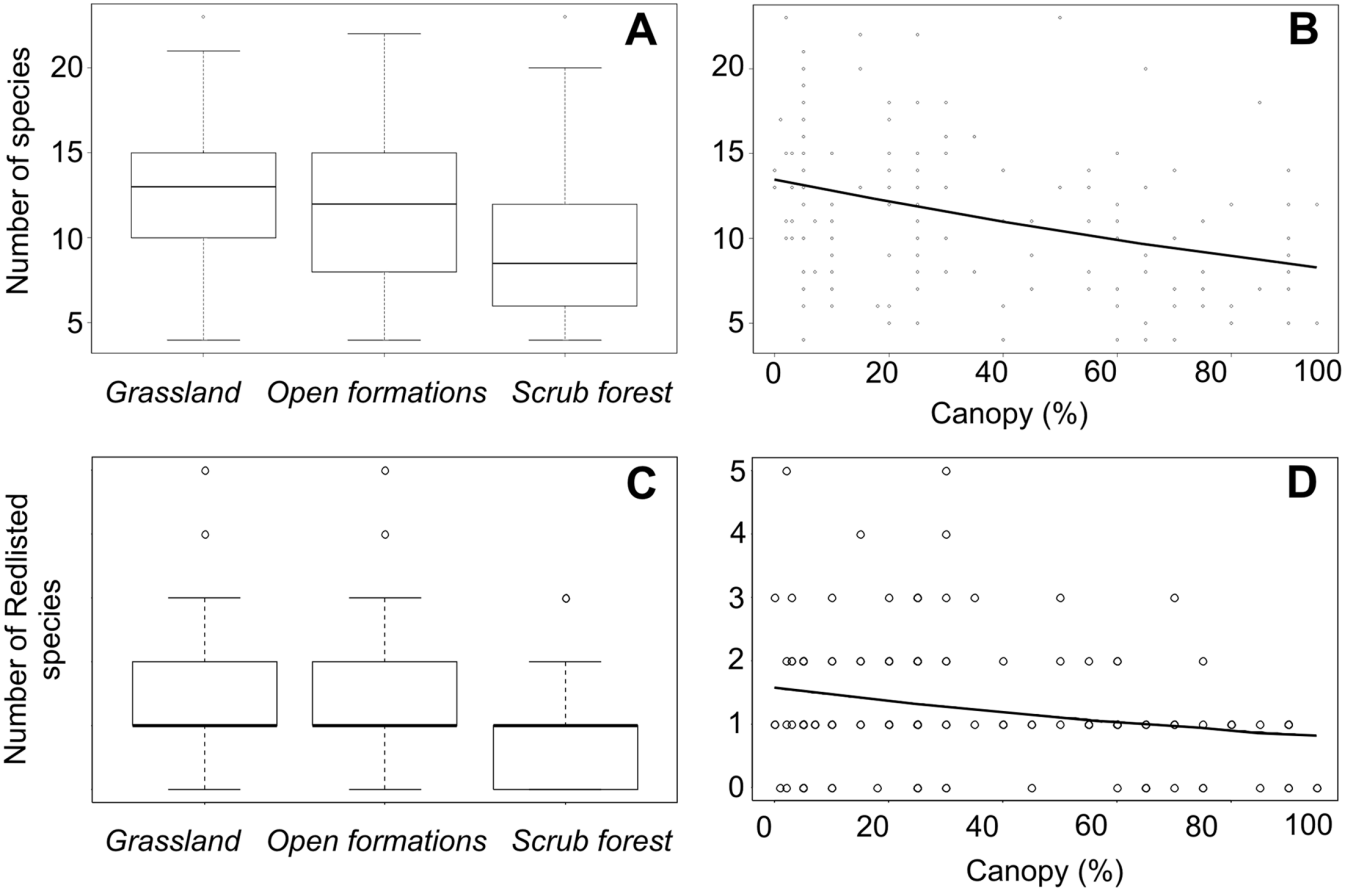
Impact of *Forest encroachment* and *Canopy* on butterfly species richness (A-B) and Red-listed species (C-D) recorded during 2012–2013 from 150 sites in the Southern Balkans. The box plots show values of species richness (A) and Red-listed species (C) predicted by the generalised linear model (glm) with *Forest Encroachment* treated as 3-level factors, no covariates included. The lines in (B–D) show glm predicted values with *Canopy* covers treated as linear predictor after inclusion of covariates for Species Richness (B) and without covariates for Red-listed species (D). See Table 1 for details.

When tested individually against all predictors and covariables, species richness responded to *Forest encroachment*, being highest in *Grasslands* and lowest in *Scrub forest*, and decreased linearly with *Canopy* cover (Fig 2A and Table 1). Of all potential covariables, *Nectar* had the strongest separate (positive) effect. Regarding site characteristics, richness was highest in intermediate longitudes, and increased with altitude. It also increased with presence of *Water* and *Herdsman’s hut*. The strongest site characteristic effect, *Veg1*, pointed to richness increasing with humidity. The combined covariate model (Table 1) explained over 38% of variation in per site species richness. Adding the predictors of interest to this model did not reveal differences among the three stages of *Forest encroachment*, but revealed a significant decline with increasing *Canopy* (Fig 2B).

**Table 1:** Results of regression models assessing the impact of forest encroachment, site environmental variables and visit circumstances on butterfly species richness in the Southern Balkans.

All species					Mediterranean species				Red listed species			
Model	AIC	DF	EV %	↓↑	AIC	DF	EV %	↓↑	AIC	DF	EV %	↓↑
**Null model (S ~ 1)**	910.93	149	100.0		566.37	149	100		410.01	149	100	
**Predictors of forest encroachment**												
S ~ *Forest encroachment*	889.11	147	9.30		567.19	147	1.73		405.13	147	6.85	
S ~ *Canopy*	881.26	148	11.40	↓	566.43	148	1.06		402.75	147	8.69	↓↓
**Site characteristics**												
S ~ *Latitude*	909.94	148	1.07	**↓↑**	503.32	148	35.41	↓	410.58	148	1.10	
S ~ *Longitude*	836.13	148	27.65	**↓↑**	560.02	148	4.54	↓	409.88	148	1.64	
S ~ *Altitude*	874.14	148	13.97	↑	561.13	148	3.94	↑	411.8	148	0.16	
S ~ *Water*	901.59	148	4.08	↑	565.03	148	1.82		411.81	148	0.15	
S ~ *Road*	909.48	148	1.24	**↑**	568.34	148	0.02		411.54	148	0.36	
S ~ *Village*	908.87	148	1.46	↓	567.53	148	0.46		411.89	148	0.09	
S ~ *Herdsman’s hut*	897.34	148	5.61	↑	559.37	148	4.90	↓	410.98	148	0.79	
S ~ *Slope*	907.46	148	1.97	↓	558.81	148	5.20	↑	410.35	148	1.28	
S ~ *Exposure*	912.25	148	0.24		568.36	148	0.01		410.38	148	1.26	
S ~ *Veg1*	802.99	148	39.58	↓	565.19	148	1.73	↑	409.67	148	1.81	
S ~ *Veg2*	909.99	148	1.06	**↓**	567.36	148	0.55		411.19	148	0.63	
S ~ *Veg3*	907.12	148	2.09	↑	567.7	148	0.36		410.73	148	0.99	
S ~ *Veg4*	911.73	148	0.43		564.84	148	1.92	↓	410.83	148	0.91	
**Visit circumstances**												
S ~ *Air Temperature*	900.33	148	4.54	↓	567.75	148	0.34		411.92	148	0.07	
S ~ *Cloudiness*	910.09	148	1.02	**↓**	560.38	148	4.35	↓	411.72	148	0.22	
S ~ *Wind*	908.05	148	1.76	↑	557.52	148	5.91	↓	410.21	148	1.39	
S ~ *Nectar*	788.35	148	44.85	↑	568.26	148	0.06		408.04	148	3.06	↑
**Covariate model (N ~ *Covariates*)**	747.46	145			499.27	146	39.80					
S ~ *Covariates + Forest Encroachment*	747.36	143	63.21		501.42	144	40.80		405.63	146	8.01	↑
S ~ *Covariates + Canopy*	744.57	144	57.74	↓	499.45	145	40.79		402.84	146	10.16	↓

Arrows indicate significant (Δ AIC ≥ 2.0) positive (↑), negative (↓), domed polynomial (↑↓) or decreasing polynomial (↓↓) response.

The covariate model, based on stepwise selection from site characteristics and visit circumstances, included the terms *Nectar + Latitude+ Altitude + Veg4*.

EV = Explained Variability, N = Number of butterfly species, S = Species Richness.

Numbers of Mediterranean species did not differ between *Forest encroachment* categories nor responded to *Canopy*. They responded to geography covariates and increased at steep *slopes* sites affected by grazing (the combined covariate model explained 39.8% of variation). Red-listed species, in contrast, responded significantly to both *Forest encroachment* (much lower in *Scrub* forest: Fig 2C) and *Canopy* (Fig 2D, polynomial decrease). On the other hand, they did not respond to any covariates except for *Nectar* (3.1% of variation). After controlling for nectar, the effects of *Forest encroachment* and *Canopy* remained significant.

### Species composition

In the single-term ordinations, both *Forest encroachment* and *Canopy* significantly affected species community composition. The explained variations were rather low, however, if compared with covariate predictors such as *Veg1*, *Nectar* or *Longitude* (Table 2). The forward selection procedure selected the following covariate model: *Altitude* + *Cloudiness* + *Latitude + Longitude + Nectar + Slope + Veg1–4 + Water + Altitude × Latitude × Longitude* (36% of variation, F = 6.3, P = 0.001). On residuals of this model, both *Forest encroachment* and *Canopy* retained their significant effects (Table 2).

**Table 2:** Results of Redundancy analyses analyzing butterfly species composition. Summary of single-term ordinations of predictors of interest (*Forest encroachment* and *Canopy)* and potential covariates (site characteristics and visit circumstances) as well as partial RDA ordinations assessing the effect of predictors of interest on butterfly species community composition (BSC) after controlling for site characteristics and visit circumstances (see Methods for details).

Null model (BSC ~ 1)	AEV	F	P
**Predictors**			
** BSC ~ *Forest encroachment***	3.00	3.30	***
** BSC ~ *Canopy***	3.30	6.20	***
**Site characteristics**			
** BSC ~ *Latitude***	6.00	10.50	***
** BSC ~ *Longitude***	10.90	19.20	***
** BSC ~ *Altitude***	4.40	7.80	***
** BSC ~ *Water***	0.60	1.90	*
** BSC ~ *Road***	0.00	0.90	
** BSC ~ *Village***	0.40	1.60	.
** BSC ~ *Herdsman’s hut***	2.50	4.80	***
** BSC ~ *Slope***	2.80	5.30	***
** BSC ~ *Exposure***	0.00	0.80	
** BSC ~ *Veg1***	12.80	22.80	***
** BSC ~ *Veg2***	2.20	4.40	***
** BSC ~ *Veg3***	1.40	3.20	**
** BSC ~ *Veg4***	2.10	4.20	***
**Visit circumstances**			
** BSC ~ *Air temperature***	2.40	4.70	***
** BSC ~ *Cloudiness***	2.20	2.90	**
** BSC ~ *Wind***	2.20	4.30	***
** BSC ~ *Nectar***	10.70	18.80	***
**Covariate model (N ~ *Covariates*)**	36.2	6.3	***
** BSC ~ *Covariates + Forest encroachment***	1.50	2.00	***
** BSC ~ *Covariates + Canopy***	1.00	2.40	***

Covariate model: BSC ~ *Altitude* + *Cloudiness* + *Latitude + Longitude + Nectar + Slope + Veg1–4 + Water + Altitude × Latitude × Longitude*.

AEV = Adjusted explanatory variable (%).

Significance as follows:

*** < 0.001;

** < 0.01;

* < 0.05;

. < 0.1.

**Fig 3:**
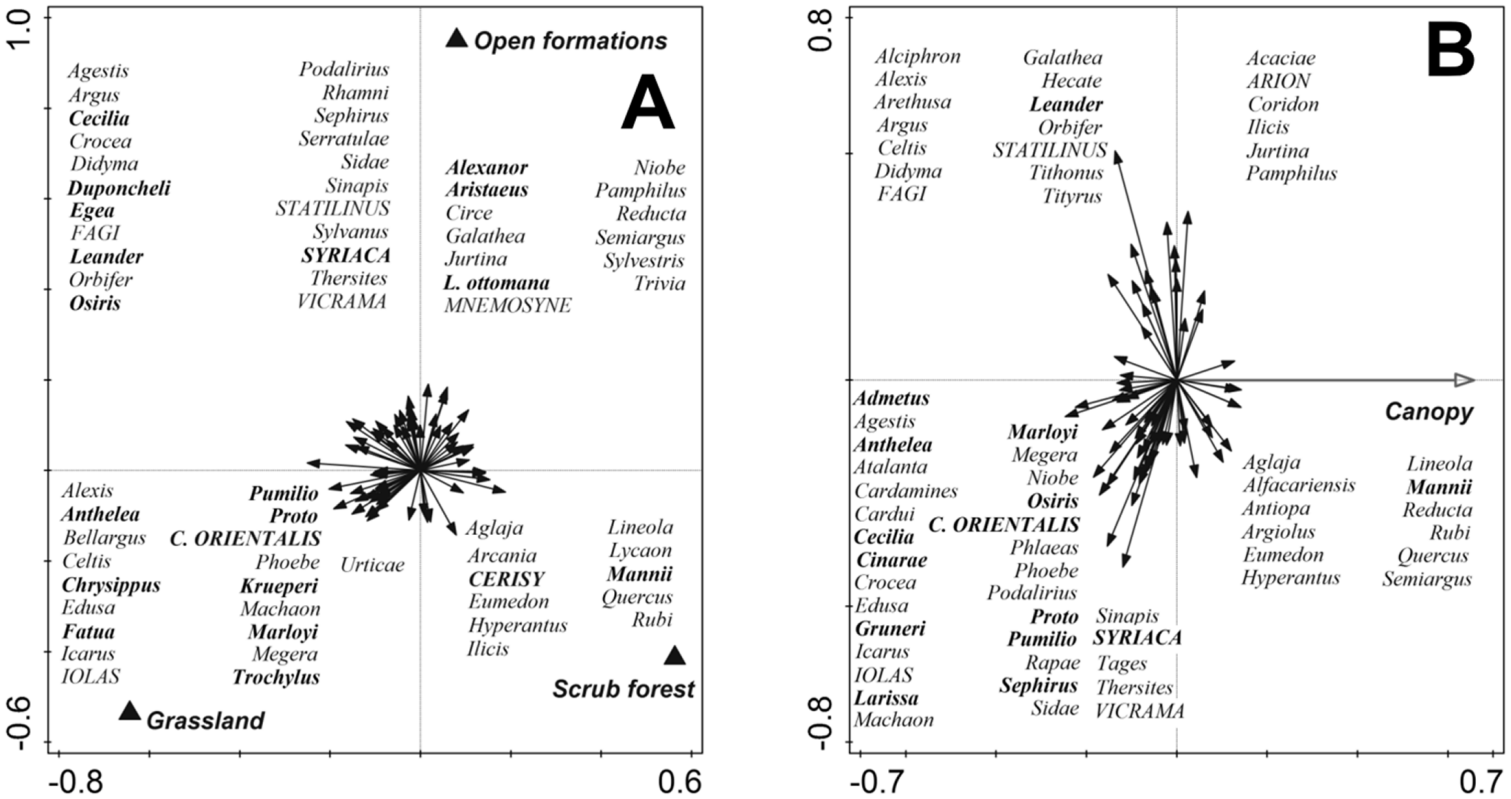
Ordination diagrams (partial redundancy analysis), showing the effect of (A) *Forest encroachment*, and (B) *Canopy* on butterfly species community composition. Both diagrams refer to analyses that statistically controlled for effects of covariates, and removed the effects of phylogeny (covariate model as in Table 2). See Table 2 for results of statistical tests. Species with Mediterranean ranges written in bold, Red-listed species in CAPITALS.

In the partial RDA with *Forest encroachment* (Fig 3A), the gradient described by the RDA axis 1 (1.67% of the variation) separated *Grasslands* from *Scrub forest*, whereas Axis 2 formed a gradient from *Grasslands/Scrub forest* towards *Open formations* (1.37%). The three forest encroachment stages thus hosted distinct sets of species. For *Grasslands*, several Mediterranean species were represented (e.g., *Pieris krueperi*, *Erynnis marloyi*), including red-listed ones (*Carcharodus orientalis*); these were accompanied by species with European or Eurosiberian distribution (e.g., *Polyommatus bellargus*, *Libythea celtis*), including the Red-listed *Iolana iolas*, and by widely distributed generalists (*Polyommatus icarus*, *Papilio machaon*). Open formations hosted distinctly high numbers of Mediterranean species (e.g., *Pyronia cecilia*. *Polygonia egea*), including Red-listed ones (*Hipparchia syriaca*), and a high number of Red-listed species with more northerly Eurosiberian distribution (*Parnassius mnemosyne*, *Hipparchia statilinus*). Finally, scrub forest hosted prevailingly non-threatened species with northerly ranges (*Argynnis aglaja*, *Aphantopus hyperantus*), although a Mediterranean representative occurred there as well (*Pieris mannii*).

In the partial RDA with *Canopy* (1.85%), practically all Mediterranean species, as well as practically all Red-listed ones, inclined towards low *Canopy* (Fig 3B).

### Species traits

Visualization of relationships among the life history traits showed a clear difference between large and mobile species with multiple generations per year, overwintering in later stages, occurring as adults late in season, having wide host plant spectra, and inhabiting large Holarctic or Eurosiberian ranges, and species with opposite traits, typically with restricted Mediterranean or European ranges (S2 Fig). The second gradient distinguished species with multiple generations, developing on forbs and/or consuming generative plant parts, from those forming few generations per year, and feeding on woody plants or grasses.

Only a few traits responded significantly to both *Forest encroachment* and *Canopy* (Table 3). *Generation numbers* responded to both predictors, indicating that closed forests were inhabited by butterflies forming fewer generations per year.

**Table 3:** Results of life history traits analysis. Traits-based interpretation of partial RDA ordinations of Southern Balkans butterfly community species composition (BSC) that assessed the response to *Forest encroachment* and *Canopy* models including significant covariates and controlled for phylogeny.

BSC ~ Response + [*Trait*] | Covariates	*Forest encroachment*			*Canopy*		
	AEV	F	P	AEV	F	P
***Altitudinal range***	0.00	0.8		0.00	0.20	
***Feeding index***	0.00	0.1		0.00	<0.1	
***Flight period***	3.40	2.0	*	0.70	1.20	
***Generation numbers***	3.90	5.9	**	3.80	5.60	*
***Gregariousness***	<0.1	1.1		1.70	3.00	
***Host plant form***	0.00	0.8		2.10	3.40	.
***Larval feeding mode***	0.50	1.6		1.90	3.30	.
***Migration***	1.20	2.5	.	0.00	0.70	
***Mountain distribution***	0.00	0.1		0.00	<0.1	
***Myrmecophily***	0.00	0.2		0.00	<0.1	
***Overwintering stage***	4.40	2.8	**	1.20	2.40	
***Ovum placement***	0.00	0.8		0.00	0.90	
***Range size***	0.00	0.9		0.20	1.2	
***Range type***	3.50	2.4	*	3.40	2.40	.
***Wingspan***	0.00	0.5		0.20	1.20	

Covariate model structure as in Table 2.

AEV = Adjusted explanatory variable (%)

Significance as follows:

** < 0.01;

* < 0.05;

. < 0.1.

**Fig 4:**
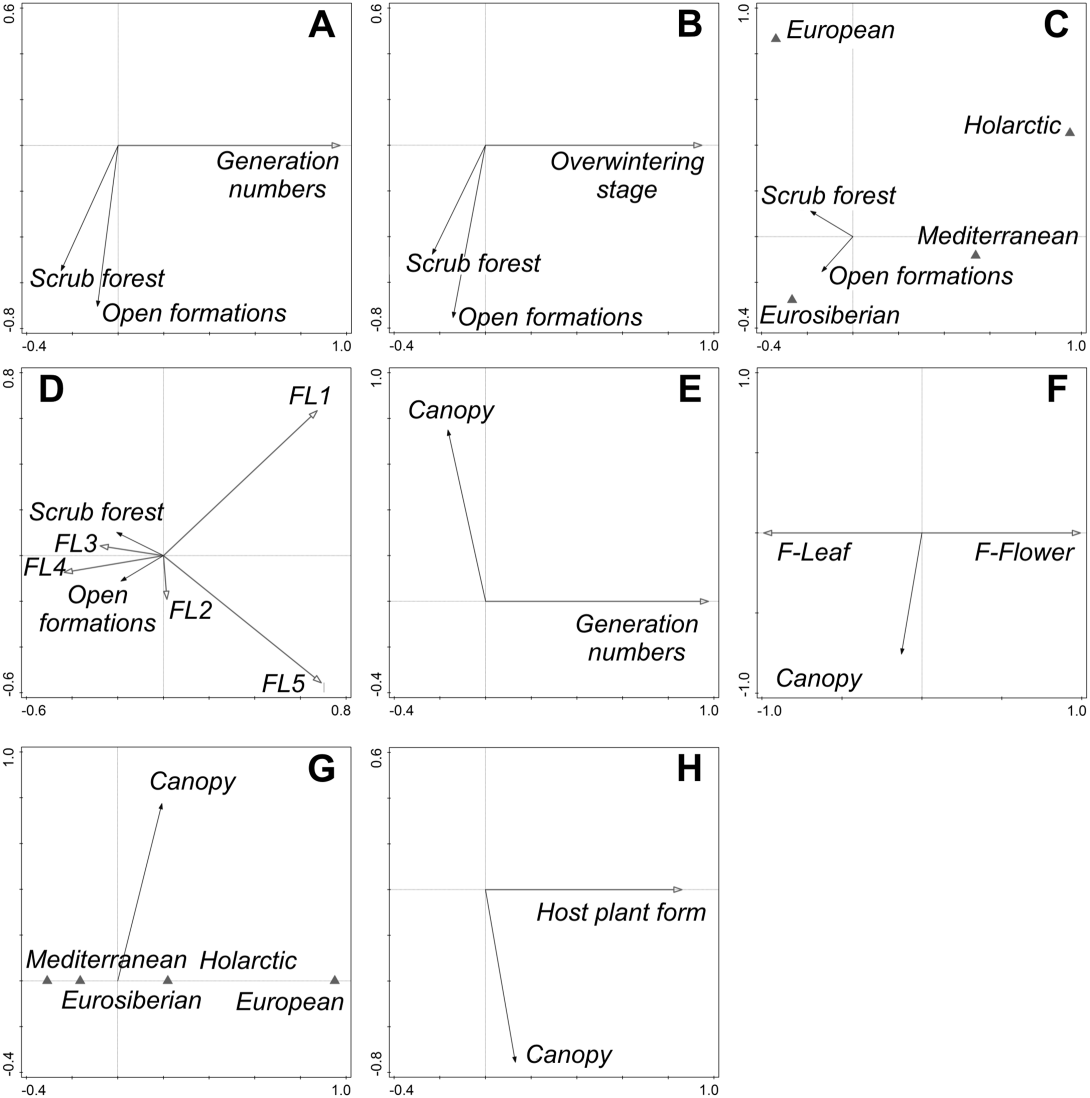
Ordination diagrams showing life-history traits interpretation of analyses of the effects of *Forest encroachment* and *Canopy* on butterfly community composition. Partial redundancy analysis, computed after including covariates (see Table 2 for formulation of covariate model) and removing the effects of phylogeny. The arrows in panels (A–D) stand for horizontal (“*Scrub forest*”) and vertical (“*Open formations*”) ordination axes in Fig 3(A), whereas panels (E–H) refer to ordination diagrams in Fig 3(B). Statistical tests in Table 3.

Regarding *Forest encroachment* (Fig 4A–4D), species overwintering in earlier stages displayed affinity towards *Scrub forest*. Vegetation closure decreased the representation of Mediterranean and Holarctic species and increased that of European and Eurosiberian species. Spring and autumn species prevailed on *Grasslands*, summer species inclined towards *Open formations* or *Scrub forest*. Regarding *Canopy* (Fig 4E–4H), there were marginally significant relationships with *Larval feeding mode* (increase of leaf chewers), *Range type* (decrease of Mediterranean and increase of Holarctic plus European species with increasing *Canopy*), and *Host plant form* (consumers of woody plants or grasses increasing with *Canopy* cover).

## Discussion

The large-scale comparison of South Balkan butterfly communities indicated that compared with grassland and open formations, sites overgrown by scrub forest hosted lower species richness and lower richness of Red-listed species, but the same number of Mediterranean species. In ordination analyses, we found profound changes in the butterfly community composition due to increasing woody vegetation cover. Interpreting these patterns using the butterflies’ life history traits showed that encroachment of formerly open landscapes by forest benefits species with fewer generations per year, overwintering in earlier stages, developing on woody plants or grasses (i.e., apparent plants, cf. [32]) and inhabiting large Eurosiberian or Holarctic ranges. It harms species forming more generations per year, developing on unapparent plants and inhabiting small Mediterranean ranges.

### Species richness and community composition along forest encroachment gradient

*Forest encroachment*, expressed either as a categorical predictor or as a proportion of woody *Canopy* cover, was associated with local butterfly richness decline regardless of the site characteristics and visit circumstances covariables for the canopy cover case. It is well known that a majority of European butterflies avoid closed-canopy habitats [33], and hence it is hardly surprising that canopy closure represents a direct threat to this insect group. Our results thus corroborate, over a relatively large geographic scale, the dependency of many butterflies occurring in the Mediterranean region on open formations (grasslands, open forests), previously documented for Mediterranean species in local-scale studies (e.g. [13, 16, 34, 35]).

Covariables increasing butterfly species richness included *Water* presence and the vegetation gradient *Veg1*, both revealing that lack of humidity restricts local species richness in the Mediterranean [36]; and *Herdsman’s hut*, suggesting positive effects of grazing-associated disturbance on species richness. *Village* proximity affected species richness negatively, indicating that species richness was not supported by other human activities than grazing. The negative effect of the vegetation covariable *Veg3* (distinguishing natural and weedy communities) supported the latter conjecture. Notably, species richness increased with altitude, which seems to contradict well known patterns of altitudinal richness decline [37, 38] but this was due to the fact that our sampling was restricted to lower elevations, not covering high mountains, while the biodiversity of the elevations in the Mediterranean seems to be drought restricted [36].

The richness patterns were strikingly different if only Mediterranean species or only Red-listed species were considered. For the former, we failed to detect a dependency on any of the two predictors describing forest encroachment. We also found meaningful responses of this group of species to potential covariates, although sometimes contrasting to those for total species richness (e.g., Mediterranean species increased, rather than decreased, with *Temperature*, and responded oppositely to the major vegetation gradient *Veg1*). For the latter, increasing canopy cover was by far the best predictor restricting their numbers, and the only significant covariate was (rather trivially) *Nectar*. These contrasting results arguably reflect definitions of the two groups. The Red-listed group contains species of all possible distribution ranges, but sharing a high degree of threat within Europe, and loss of open habitats threatens European butterflies in general [28, 39]. In contrast, the Mediterranean group is defined by shared distribution range, independently of habitats, and our samples included species of all possible habitats, from bare grounds (e.g., *Carchardodus orientalis*, *Chilades trochylus*, *Pseudochazara anthelea*) to closed forest (*Kirinia roxelana*, *Zerynthia cerisy*) (cf. [40]). Thus, apart from the low number of Red-listed species in closed canopy sites, analysing mere species numbers does not convey much information regarding individual species requirements.

The ordination analyses focusing on species composition provided deeper insights. Treating forest encroachment levels as a categorical predictor showed that each of the three categories hosted some Mediterranean and some Red-listed species, although both *Grasslands* and *Open formations* hosted apparently more such species than *Scrub forest*. Moreover, each of the two categories attracted distinct species, suggesting that to sustain the whole butterfly diversity associated with traditional Mediterranean landscapes, mosaics of alteration of grasslands open “savannah-like” formations are necessary. Note that even open habitat species may temporarily utilise cooler microclimates provided by close canopy sites [41], which explains our scrub forest records of such species as *Hipparchia statilinus*, a Mediterranean species requiring near-bare ground for larval development (27 forest presence records out of 85) (cf. [42]). Open formations hosted both Mediterranean species, some of them threatened (e.g., *Hipparchia syriaca*), in combination with species that prefer barren surfaces in more northerly parts of their ranges (e.g., *Hipparchia statilinus*, *Pseudopilotes vicrama*) [28, 43].

The patterns found for the numeric predictor *Canopy* were even clearer, revealing avoidance of Mediterranean species, and affinity of northern species, towards increasing *Canopy* cover.

### Species traits changes along forest encroachment

The prevailingly European species colonising *Scrub forest* sites form few generations per year, overwinter in early stages, and, counter-intuitively, fly in spring; the prevailingly Eurosiberian species colonising *Open formations* fly mainly in high summer; and the prevailingly Mediterranean species colonising *Grasslands* were mainly spring or autumn flying species. It follows that a link exists between species ranges, habitat successional stage and associated butterflies’ development. This was previously suggested by Dennis et al. [21], who related the life histories of British butterflies to the life history strategies of their host plants. Association of slowly developing species forming few generations per annum with late successional habitats has been reported from such disparate regions as Germany [44], Catalonia [45] and Japan [46]. This is sometimes attributed to habitat disturbance dynamics, in that rarely disturbed habitats allow for slower insect reproductive rates in contrast to frequently disturbed habitats. This interpretation, however, fails to explain why woodland species *both* overwinter in early stages and occur as adults early in spring, which forces them to develop rapidly. An alternative explanation, suggested, e.g. by Cizek et al. [32] invokes the nature of antiherbivore defenses in late-successional plants (trees, coarse grasses). In such plants, quantitative defenses (tannin, silica et.) prevail, restricting associated herbivores’ development to young plant tissues, available in early season. In parallel, woodland species developing on forbs are constrained to early development by rapid canopy shading, or progressive host plant senescence (e.g. [47]). The marginally significant effect of host plant form in our analysis circumstantially supports the plant defenses role. Moreover, species with higher generation numbers and species overwintering in later stages inclined towards grasslands, where the combined effects of host plants senescence and canopy shading do not apply.

Similar logic may explain the link between Mediterranean distribution, spring plus autumn adult period, and *Grasslands*. Grasslands receive enough sun early in the year, get hot and dry during high summer, but become inhabitable again with autumn rains [48]. Then, multivoltine species (e.g. *Gegenes pumilio*, *Pieris krueperi*, *Chilodes trochylus*) form additional generation(s), whereas univoltine species with long-living adults (cf. [49]) locate both nectar and oviposition substrates there. The association of species flying in high summer with *Open formations* is best explained by the structural heterogeneity of such sites, where mosaics of closed and open vegetation offer varying microclimate conditions, supplying some nectar, moisture and shade even during summer.

### Conservation Implications

Species’ ranges result from phylogenetic history, dispersal and habitat requirements [50]. The avoidance of closed canopy sites by the range-restricted Mediterranean species, and their affinity for either *Open formations* or *Grasslands*, agrees with results recently reported for Greek birds [51] and spiders [14]. Grill et al. [16] and Kati et al. [12] reported, for butterflies and orthopterans, respectively, the highest species richness, and highest representations of range-restricted species, from such richly structured habitats as abandoned orchards and wooded pastures in the Greek nature reserve Dadia. Increases of common northern species at the expense of Mediterranean endemics were also detected for southern French birds [52], Sardinian plants [53], and Catalonian (i.e., West Mediterranean) butterflies [15]. Assuming historical conservatism of species life histories, the negative association of Mediterranean species with closed canopy condition falsifies the “forested Mediterranean” hypothesis, highlighting the need to maintain open landscapes across the region.

Notably, the increases of northern species due to forest encroachment contradict the predictions that northern species should decline at their southern range margins due to the current climatic warming [54]. This process is probably counteracted by another development, detected for Greek butterflies by Zografou et al. [55], who found an increase of low-altitude thermophilous species against high-altitude ones. The two processes, increase in the representation of thermophilous species due to warming climate and their decrease due to habitat loss, are likely affecting species individually, depending on their ability to adapt, e.g. by locating sites with suitable microclimates [56]. For global conservation, however, the outcomes are hardly positive, because the majority of the Mediterranean endemics depend on grasslands or open formations, habitats that are rapidly decreasing all over the study region.

Without maintaining rich mosaics of open and semi-open habitats across the southern Balkans, the restructuring of butterfly and other small animal communities due to forest encroachment will gradually replace range-restricted endemic fauna by wide ranging generalists. Maintaining open landscapes is complicated by several factors. First, such widely advocated land management tools as “headage payments” for shepherds [8] or agro-environmental schemes rewarding environmentally benign farming [57], were originally designed in north-western Europe and may be poorly transferable to the conditions of Southern Europe, with much more diverse habitat conditions and declining rural population [58]. Second, financial incentives do not guarantee that human impacts on habitats replicate those existing in the past. For instance, agrotechnology developments such as fodder crops production and vehicle transport relaxed the need to harvest summer coppice, or to move herds across the landscapes (transhumance) (cf. [59]). Cizek et al. [60] documented that current management technologies fail to provide microhabitat heterogeneity needed for reserve management in Central Europe, and the outcomes may be even worse in species-richer Southern Europe. Still worse, relying on subsidies assumes constant economic growth, which is far from guaranteed in the long term. Economic decline might promote returns of urban population to villages, but this would be a long-term process, whereas breakdowns in funding may lead to rapid habitat and species losses.

Without downplaying the subsidised efforts to maintain rural habitats diversity [61], novel approaches which would maintain the open to semi-open conditions across the Mediterranean while being economically sustainable should be sought. At least locally, declining grazing by farm animals might be replaced by free ranging ungulates, including species historically extirpated from the Mediterranean [62]. Such projects would, at least regionally, return to the Mediterranean the key players that had been affecting ecosystem dynamics before the advent of farming, and with which the regionally endemic biodiversity has evolved.

## Supporting Information

S1 Fig Description of the direct ordination used to extract vegetation variables from species composition of the sites. (DOCX)

S2 Fig Unconstrained analysis of butterfly species life history traits. (DOCX)

S1 Raw Data Raw data used for analysing butterfly species richness and community composition. (XLSX)

S1 Table Checklist of butterfly species recorded in individual regions and total numbers of records (+/–indicate presence/absence). (DOCX)

S2 Table List of butterfly species life-history traits used to analyse impacts of forest encroachment on South Balkans butterflies, associated hypotheses and relevant references. (DOCX)

S3 Table List of life history traits, used to analyse impacts of forest encroachment on South Balkans butterflies. (DOCX)

S1 Text Detailed description of study regions, south Balkans, 2013–2014. (DOCX)

S2 Text Reference list to the sources of phylogenetic information. (DOCX)

## References

[1] Myers N, Mittermeier RA, Mittermeier CG, da Fonseca GAB, Kent J. Biodiversity hotspots for conservation priorities. Nature. 2000;403:853–8. 10.1038/35002501 10706275

[2] Metzger MJ, Bunce RGH, Jongman RHG, Mucher CA, Watkins JW. A climatic stratification of the environment of Europe. Global Ecology and Biogeography. 2005;14:549–63. 10.1111/j.1466-822x.2005.00190.x

[3] Blondel J, Aronson J. Biology and wildlife in the Mediterranean region. Oxford: Oxford University Press; 1999.

[4] Grove AT, Rackham O. The Nature of Mediterranean Europe—An Ecological History. New Haven: Yale University Press; 2003.

[5] Van Dyck H, Van Strien AJ, Maes D, Van Swaay CAM. Declines in Common, Widespread Butterflies in a Landscape under Intense Human Use. Conservation Biology. 2009;23:957–65. 10.1111/j.1523-1739.2009.01175.x 19637406

[6] MacDonald D, Crabtree JR, Wiesinger G, Dax T, Stamou N, Fleury P, et al Agricultural abandonment in mountain areas of Europe: Environmental consequences and policy response. J Environ Manage. 2000;59:47–69. 10.1006/jema.1999.0335

[7] Strijker D. Marginal lands in Europe—causes of decline. Basic and Applied Ecology. 2005;6:99–106. 10.1016/j.baae.2005.01.001

[8] Stoate C, Baldi A, Beja P, Boatman ND, Herzon I, van Doorn A, et al Ecological impacts of early 21st century agricultural change in Europe—A review. J Environ Manage. 2009;91:22–46. 10.1016/j.jenvman.2009.07.005 19717221

[9] Debussche M, Lepart J, Dervieux A. Mediterranean landscape changes: evidence from old postcards. Global Ecology and Biogeography. 1999;8:3–15. 10.1046/j.1365-2699.1999.00316.x

[10] Dover JW, Rescia A, Fungarino S, Fairburn J, Carey P, Lunt P, et al Can hay harvesting detrimentally affect adult butterfly abundance? Journal of Insect Conservation. 2010;14:413–8. 10.1007/s10841-010-9267-5

[11] Hadjigeorgiou I, Osoro K, de Almeida JPF, Molle G. Southern European grazing lands: Production, environmental and landscape management aspects. Livest Prod Sci. 2005;96:51–9. 10.1016/j.livprodsci.2005.05.016

[12] Kati V, Dufrene M, Legakis A, Grill A, Lebrun P. Conservation management for Orthoptera in the Dadia reserve, Greece. Biological Conservation. 2004;115:33–44. 10.1016/S0006-3207(03)00091-0

[13] Grill A, Knoflach B, Cleary DFR, Kati V. Butterfly, spider, and plant communities in different land-use types in Sardinia, Italy. Biodivers Conserv. 2005;14:1281–300. 10.1007/s10531-004-1661-4

[14] Zakkak S, Chatzaki M, Karamalis N, Kati V. Spiders in the context of agricultural land abandonment in Greek Mountains: species responses, community structure and the need to preserve traditional agricultural landscapes. Journal of Insect Conservation. 2014;18:599–611. 10.1007/s10841-014-9663-3

[15] Stefanescu C, Torre I, Jubany J, Paramo F. Recent trends in butterfly populations from north-east Spain and Andorra in the light of habitat and climate change. Journal of Insect Conservation. 2011;15:83–93. 10.1007/s10841-010-9325-z

[16] Grill A, Cleary DFR. Diversity patterns in butterfly communities of the Greek nature reserve Dadia. Biological Conservation. 2003;114:427–36. 10.1016/s0006-3207(03)00070-3

[17] Sirami C, Brotons L, Martin JL. Vegetation and songbird response to land abandonment: from landscape to census plot. Divers Distrib. 2007;13:42–52. 10.1111/j.1472-4642.2006.00297.x

[18] Cremene C, Groza G, Rakosy L, Schileyko AA, Baur A, Erhardt A, et al Alterations of steppe-like grasslands in Eastern Europe: a threat to regional biodiversity hotspots. Conservation Biology. 2005;19:1606–18. 10.1111/j.1523-1739.2005.00084.x

[19] Stefanescu C, Carnicer J, Penuelas J. Determinants of species richness in generalist and specialist Mediterranean butterflies: the negative synergistic forces of climate and habitat change. Ecography. 2011;34:353–63. 10.1111/j.1600-0587.2010.06264.x

[20] Carnicer J, Stefanescu C, Vila R, Dinca V, Font X, Penuelas J. A unified framework for diversity gradients: the adaptive trait continuum. Global Ecology and Biogeography. 2013;22:6–18. 10.1111/j.1466-8238.2012.00762.x

[21] Dennis RLH, Hodgson JG, Grenyer R, Shreeve TG, Roy DB. Host plants and butterfly biology. Do host-plant strategies drive butterfly status? Ecol Entomol. 2004;29:12–26. 10.1111/j.1365-2311.2004.00572.x

[22] McGill BJ, Enquist BJ, Weiher E, Westoby M. Rebuilding community ecology from functional traits. Trends Ecol Evol. 2006;21:178–85. 10.1016/j.tree.2006.02.002 16701083

[23] Borschig C, Klein AM, von Wehrden H, Krauss J. Traits of butterfly communities change from specialist to generalist characteristics with increasing land-use intensity. Basic and Applied Ecology. 2013;14:547–54. 10.1016/j.baae.2013.09.002

[24] Bartonova A, Benes J, Konvicka M. Generalist-specialist continuum and life history traits of Central European butterflies (Lepidoptera)—are we missing a part of the picture? European Journal of Entomology. 2014;111:543–53. 10.14411/eje.2014.060

[25] Van Swaay CAM, Nowicki P, Settele J, Van Strien AJ. Butterfly monitoring in Europe: methods, applications and perspectives. Biodivers Conserv. 2008;17:3455–69. 10.1007/s10531-008-9491-4

[26] Kadlec T, Tropek R, Konvicka M. Timed surveys and transect walks as comparable methods for monitoring butterflies in small plots. Journal of Insect Conservation. 2012;16:275–80. 10.1007/s10841-011-9414-7

[27] Fauna Europaea version 2.6. [Internet]. 2013 [cited 7 July 2014]. Available from: http://www.faunaeur.org.

[28] Van Swaay CAM, Collins S, Maes D, Lopez Munguira M, Sasic M, Settele J, et al European Red List of Butterflies. Luxembourg: Publications Office of the European Union; 2010.

[29] Tansley AG, Chip TF. Aims and Methods in Study of Vegetation. London: Whitefriars; 1926.

[30] The R Core Team. R version 2. 15. 2. 2012 [cited 2013 September 10]. Available from: http://www.r-project.org.

[31] Ter Braak CJF, Smilauer P. Canoco 5, Windows release (5.00) 2013 [cited 2014 February 5, 2014]. Available from: www.canoco5.com.

[32] Cizek L, Fric Z, Konvicka M. Host plant defences and voltinism in European butterflies. Ecol Entomol. 2006;31:337–44. 10.1111/j.1365-2311.2006.00783.x

[33] Settele J, Shreeve T, Konvička M, Van Dyck H. Part 5 Global Change and Conservation In: Settele J, Shreeve T, Konvička M, Van Dyck H, editors. Ecology of Butterflies in Europe. Cambridge: Cambridge University Press; 2009 p. 315–70.

[34] Zografou K, Sfenthourakis S, Pullin A, Kati V. On the surrogate value of red-listed butterflies for butterflies and grasshoppers: a case study in Grammos site of Natura 2000, Greece. Journal of Insect Conservation. 2009;13:505–14. 10.1007/s10841-008-9198-6

[35] Slancarova J, Garcia-Pereira P, Fric Z, Romo H, Garcia-Barros E. Butterflies in Portuguese ‘montados’: relationships between climate, land use and life-history traits. Journal of Insect Conservation. 2015:1–14. 10.1007/s10841-015-9801-6

[36] Stefanescu C, Herrando S, Paramo F. Butterfly species richness in the north-west Mediterranean Basin: the role of natural and human-induced factors. Journal of Biogeography. 2004;31:905–15. 10.1111/j.1365-2699.2004.01088.x

[37] Leingartner A, Krauss J, Steffan-Dewenter I. Species richness and trait composition of butterfly assemblages change along an altitudinal gradient. Oecologia. 2014;175:613–23. 10.1007/s00442-014-2917-7 24668013

[38] Sanchez-Rodriguez JF, Baz A. The effects of elevation on the butterfly communities of a Mediterranean mountain, Sierra de Javalambre, central Spain. Journal of the Lepidopterists' Society. 1995;49:192–207.

[39] van Swaay C, van Strien A, Harpke A, Fontaine B, Stefanescu C, Roy D, et al. The European grassland butterfly indicator: 1990–2011. EEA Technical report. 2013.

[40] Pamperis LN. The butterflies of Greece. Athens: Bastas-Plessas Graphic Arts S.A.; 1997.

[41] Slamova I, Klecka J, Konvicka M. Woodland and grassland mosaic from a butterfly perspective: habitat use by Erebia aethiops (Lepidoptera: Satyridae). Insect Conserv Diver. 2013;6:243–54. 10.1111/j.1752-4598.2012.00212.x

[42] Steiner R, Trusch R. Eiablageverhalten und habitat von Hipparchia statilinus in Brandenburg (Lepidoptera: Nymphalidae: Satyrinae). Stuttgarter Beitr Naturk A Biol. 2000;606:1–10.

[43] Tolman T, Lewington R. Collins Butterfly Guide: The Most Complete Guide To The Butterflies Of Britain And Europe. London: HarperCollins; 2009.

[44] Steffan-Dewenter I, Tscharntke T. Early succession of butterfly and plant communities on set-aside fields. Oecologia. 1997;109:294–302. 10.1007/s004420050087 28307183

[45] Stefanescu C, Penuelas J, Filella I. Butterflies highlight the conservation value of hay meadows highly threatened by land-use changes in a protected Mediterranean area. Biological Conservation. 2005;126:234–46. 10.1016/j.biocon.2005.05.010

[46] Ohwaki A, Nakamura K, Tanabe SI. Butterfly assemblages in a traditional agricultural landscape: importance of secondary forests for conserving diversity, life history specialists and endemics. Biodivers Conserv. 2007;16:1521–39. 10.1007/s10531-006-9042-9

[47] Konvicka M, Kuras T. Population structure, behaviour and selection of oviposition sites of an endangered butterfly, Parnassius mnemosyne, in Litovelske Pomoravi, Czech Republic. Journal of Insect Conservation. 1999;3:211–23. 10.1023/a:1009641618795

[48] Lopez-Villalta JS. Ecological trends in endemic Mediterranean butterflies. Bull Insectology. 2010;63:161–70.

[49] Garcia-Barros E. Delyed ovarian maturation in the butterfly *Hipparchia-semele* as a possible response to summer drought. Ecol Entomol. 1988;13:391–8. 10.1111/j.1365-2311.1988.tb00371.x

[50] Schoville SD, Roderick GK. Alpine biogeography of Parnassian butterflies during Quaternary climate cycles in North America. Molecular Ecology. 2009;18:3471–85. 10.1111/j.1365-294X.2009.04287.x 19659481

[51] Zakkak S, Kakalis E, Radovic A, Halley JM, Kati V. The impact of forest encroachment after agricultural land abandonment on passerine bird communities: The case of Greece. Journal for Nature Conservation. 2014;22:157–65. 10.1016/j.jnc.2013.11.001

[52] Coreau A, Martin JL. Multi-scale study of bird species distribution and of their response to vegetation change: a Mediterranean example. Landsc Ecol. 2007;22:747–64. 10.1007/s10980-006-9074-2

[53] Farris E, Filigheddu R, Deiana P, Farris GA, Garau G. Short-term effects on sheep pastureland due to grazing abandonment in a Western Mediterranean island ecosystem: A multidisciplinary approach. Journal for Nature Conservation. 2010;18:258–67. 10.1016/j.jnc.2009.11.003

[54] Parmesan C. Ecological and evolutionary responses to recent climate change Annual Review of Ecology Evolution and Systematics. Annual Review of Ecology Evolution and Systematics. 37 Palo Alto: Annual Reviews; 2006 p. 637–69.

[55] Zografou K, Kati V, Grill A, Wilson RJ, Tzirkalli E, Pamperis LN, et al Signals of Climate Change in Butterfly Communities in a Mediterranean Protected Area. PLoS One. 2014;9 10.1371/journal.pone.0087245 PMC3906159 24489880

[56] Suggitt AJ, Stefanescu C, Paramo F, Oliver T, Anderson BJ, Hill JK, et al Habitat associations of species show consistent but weak responses to climate. Biol Lett. 2012;8:590–3. 10.1098/rsbl.2012.0112 22491762 PMC3391465

[57] Knop E, Kleijn D, Herzog F, Schmid B. Effectiveness of the Swiss agri-environment scheme in promoting biodiversity. Journal of Applied Ecology. 2006;43:120–7. 10.1111/j.1365-2664.2005.01113.x

[58] Bunce RGH, Bell M, Farino T. The environmentally sensitive area legislation in the United Kingdom and its potential application to the Picos de Europa mountains in north-west Spain. Environmental Conservation. 1998;25:219–27. 10.1017/s0376892998000277

[59] Van der Leeuw S. Vegetation Dynamics and Land Use in Epirus Recent Dynamics of the Mediterranean Vegetation and Landscape: John Wiley & Sons, Ltd; 2004 p. 121–41.

[60] Cizek O, Zamecnik J, Tropek R, Kocarek P, Konvicka M. Diversification of mowing regime increases arthropods diversity in species-poor cultural hay meadows. Journal of Insect Conservation. 2012;16:215–26. 10.1007/s10841-011-9407-6

[61] Dover JW, Rescia A, Fungarino S, Fairburn J, Carey P, Lunt P, et al Land-use, environment, and their impact on butterfly populations in a mountainous pastoral landscape: species richness and family-level abundance. Journal of Insect Conservation. 2011;15:523–38. 10.1007/s10841-010-9331-1

[62] Dobson M. Mammal distributions in the western Mediterranean: the role of human intervention. Mammal Review. 1998;28:77–88. 10.1046/j.1365-2907.1998.00027.x

