# Supplementary material for: Life History Traits Reflect Changes in Mediterranean Butterfly Communities Due to Forest Encroachment
Source: PLoS One. 2016 Mar 21;11(3):e0152026. doi: 10.1371/journal.pone.0152026 (PMC4801352; doi:10.1371/journal.pone.0152026)
Supplement: S1 Fig — (DOCX) [file pone.0152026.s001.docx]

**Life History Traits Reflect Changes in Mediterranean Butterfly Communities due to Forest Encroachment**

**Short title: Forest Encroachment and Mediterranean Butterflies**

Jana Slancarova^1,2*^, Alena Bartonova^1,2^, Michal Zapletal^1,2^, Milan Kotilinek^1^, Zdenek Faltynek Fric^2^, Nikola Micevski^3^, Vasiliki Kati^4^, Martin Konvicka^1,2*^

^1^ Faculty of Science, University of South Bohemia, Ceske Budejovice, Czech Republic

^2^ Institute of Entomology, Biology Centre CAS, Ceske Budejovice, Czech Republic

^3^ Macedonian Entomological Society (ENTOMAK), Skopje, Republic of Macedonia (FYROM)

^4^ Department of Environmental and Natural Resources Management, University of Patras,

Agrinio, Greece

^*^ corresponding authors, emails: konva333@gmail.com (MK), slancaro@mail.com (JS)

**S1 Figure. Description of the direct ordination used to extract vegetation variables from species composition of the sites.**

The vegetation community composition of the 150 recording sites was compared using a detrended canonical correspondence analysis (DCA), an unconstrained ordination method (computed in CANOCO 5.0; Ter Braak and Smilauer, 2013), on log-transformed plant species abundances.

The ordination returned four ordination axes, i.e. four novel predictors used to analyse the butterfly records. These were: *Veg1* (corresponding to decreasing humidity), *Veg2* (increasing representation of woody plants), *Veg3* (distinguishing abandoned fields from natural vegetation), and *Veg4* (indication of oligotrophic/eutrophic gradient). For statistics details see Table B1. Ordination diagrams are shown on Fig. B1 and Fig. B2, see Table B2 for abbreviated plant names.

**Table B1.** Statistics summary table, total variation 20.888.

| **Statistics** | **Axis 1** | **Axis 2** | **Axis 3** | **Axis 4** |
| --- | --- | --- | --- | --- |
| Eigenvalues | 0.574 | 0.418 | 0.335 | 0.291 |
| Explained variation (cumulative) | 2.75 | 4.75 | 6.35 | 7.75 |
| Gradient length | 5.17 | 3.97 | 3.74 | 3.52 |


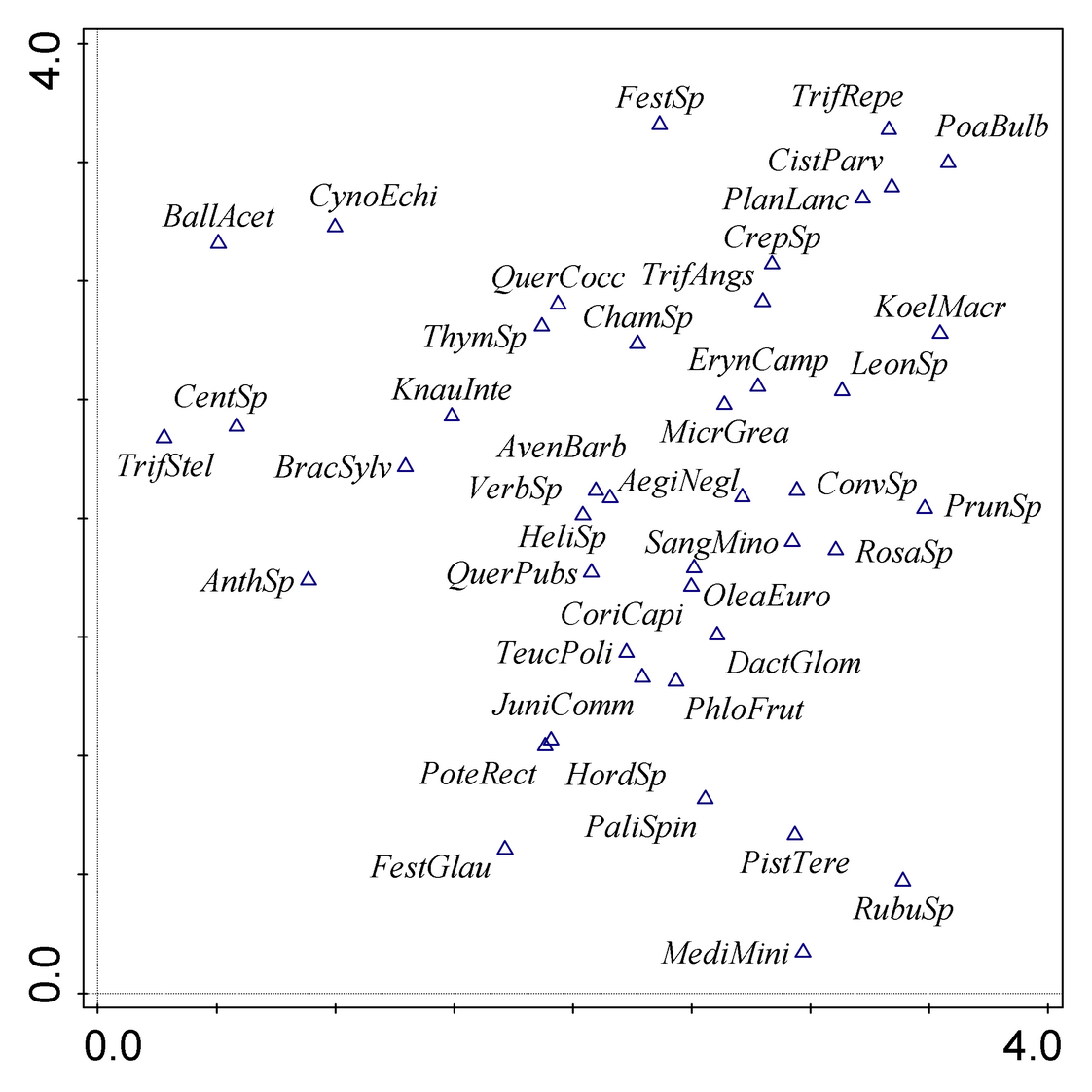


**Figure B1.** DCA ordination diagram showing first and second ordination axes


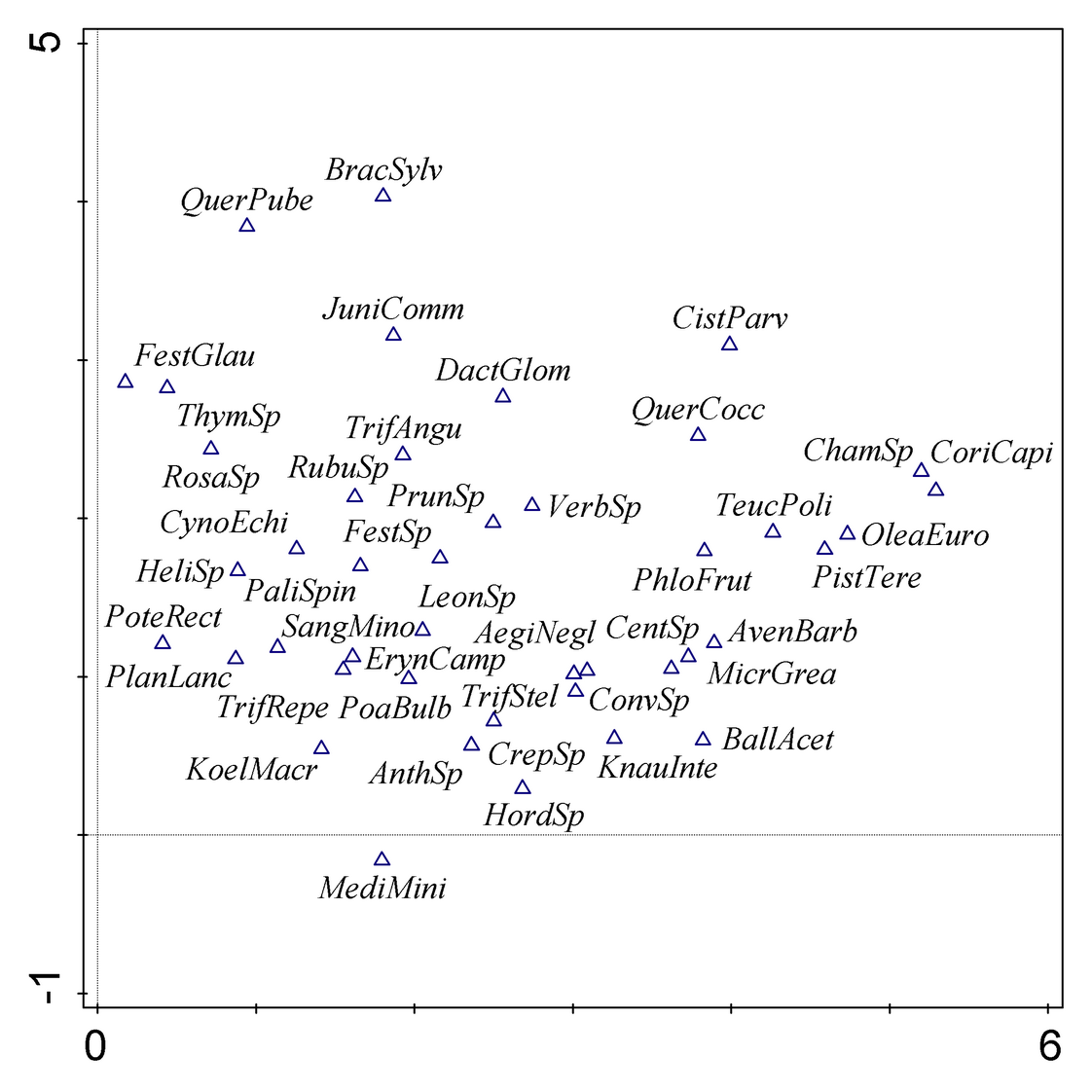


**Figure B2.** DCA ordination diagram showing third and fourth ordination axes

**Table B2.** Abbreviations of plants depicted in the ordination diagrams (Fig. B1 and Fig. B2).

| ***Abbreviation*** | ***Genus*** | ***Species*** |
| --- | --- | --- |
| *AegiNegl* | *Aegilops* | *neglecta* |
| *AnthSp* | *Anthemis* | *sp.* |
| *AvenBarb* | *Avena* | *barbata* |
| *BallAcet* | *Ballota* | *acetabulosa* |
| *BracSylv* | *Brachypodium* | *sylvaticum* |
| *CentSp* | *Centaurea* | *sp.* |
| *CistParv* | *Cistus* | *parviflorus* |
| *ConvSp* | *Convolvulus* | *sp.* |
| *CoriCapi* | *Coricothymus* | *capitatus* |
| *CrepSp* | *Crepis* | *sp.* |
| *CynoEchi* | *Cynosorus* | *echinatus* |
| *DactGlom* | *Dactylis* | *glomerata* |
| *ErynCamp* | *Eryngium* | *campestre* |
| *FestPall* | *Festuca* | *pallens* |
| *FestSp* | *Festuca* | *sp.* |
| *HeliSp* | *Helianthemum* | *sp.* |
| *HordSp* | *Hordeum* | *sp.* |
| *ChamSp* | *Chamaecytisus* | *sp.* |
| *JuniComm* | *Juniperus* | *communis* |
| *KnauInte* | *Knautia* | *integrifolia* |
| *KoelMacr* | *Koeleria* | *macrantha* |
| *LeonSp* | *Leontodon* | *sp.* |
| *MediMini* | *Medicago* | *minima* |
| *MicrGrea* | *Micromeria* | *greaca* |
| *OleaEuro* | *Olea* | *europea* |
| *PaliSpin* | *Paliorus* | *sp.ina-christi* |
| *PhloFrut* | *Phlomis* | *fruticosa* |
| *PistTere* | *Pistacia* | *terebinthus* |
| *PlanLanc* | *Plantago* | *lanceolata* |
| *PoaBulb* | *Poa* | *bulbosa* |
| *PoteRect* | *Potentila* | *recta* |
| *PrunSp* | *Prunus* | *sp.* |
| *QuerCocc* | *Quercus* | *coccifera* |
| *QuerPube* | *Quercus* | *pubescens* |
| *RosaSp* | *Rosa* | *sp.* |
| *RubuSp* | *Rubus* | *sp.* |
| *SangMino* | *Sanguisorba* | *minor* |
| *TeucPoli* | *Teucrium* | *polium* |
| *ThymSp* | *Thymus* | *sp.* |
| *TrifAngu* | *Trifolium* | *angustifolium* |
| *TrifRepe* | *Trifolium* | *repens* |
| *TrifStel* | *Trifolium* | *stellatum* |
| *VerbSp* | *Verbascum* | *sp.* |

**References**

Ter Braak CJF, Smilauer P. Canoco 5, Windows release (5.00) 2013 [cited 2014 February 5, 2014]. Available from: www.canoco5.com.
