# Supplementary material for: Life History Traits Reflect Changes in Mediterranean Butterfly Communities Due to Forest Encroachment
Source: PLoS One. 2016 Mar 21;11(3):e0152026. doi: 10.1371/journal.pone.0152026 (PMC4801352; doi:10.1371/journal.pone.0152026)
Supplement: S2 Fig — (DOCX) [file pone.0152026.s002.docx]

**Life History Traits Reflect Changes in Mediterranean Butterfly Communities due to Forest Encroachment**

**Short title: Forest Encroachment and Mediterranean Butterflies**

Jana Slancarova^1,2*^, Alena Bartonova^1,2^, Michal Zapletal^1,2^, Milan Kotilinek^1^, Zdenek Faltynek Fric^2^, Nikola Micevski^3^, Vasiliki Kati^4^, Martin Konvicka^1,2*^

^1^ Faculty of Science, University of South Bohemia, Ceske Budejovice, Czech Republic

^2^ Institute of Entomology, Biology Centre CAS, Ceske Budejovice, Czech Republic

^3^ Macedonian Entomological Society (ENTOMAK), Skopje, Republic of Macedonia (FYROM)

^4^ Department of Environmental and Natural Resources Management, University of Patras,

Agrinio, Greece

^*^ corresponding authors, emails: konva333@gmail.com (MK), slancaro@mail.com (JS)

**S2 Figure. Unconstrained analysis of butterfly species life history traits.**

Butterfly life-history traits were compared using a principal correspondence analysis (PCA), unconstrained ordination method, in CANOCO 5.0 [1]. Individual species formed “samples” and their traits formed “species data”, in CANOCO notations.

It is apparent from Fig. 1 that the first ordination axis differentiated between large, mobile and multivoltine “generalists” inhabiting large ranges (positive values on the axis) and small, monovoltine, dense populations forming “specialists”, typically inhabiting small ranges (negative values of the axis). On the second axis, there is a gradient distinguishing species with multiple generations, developing on unapparent plants and/or consuming generative plant parts (positive values), from those feeding on apparent host plants and having few generations per year (negative values).

**Table C1.** Statistics summary table, total variation 4128.00.

| **Statistic** | **Axis 1** | **Axis 2** | **Axis 3** | **Axis 4** |
| --- | --- | --- | --- | --- |
| Eigenvalues | 0.1638 | 0.1245 | 0.0876 | 0.0736 |
| Explained variation (cumulative) | 16.38 | 28.83 | 37.59 | 44.95 |


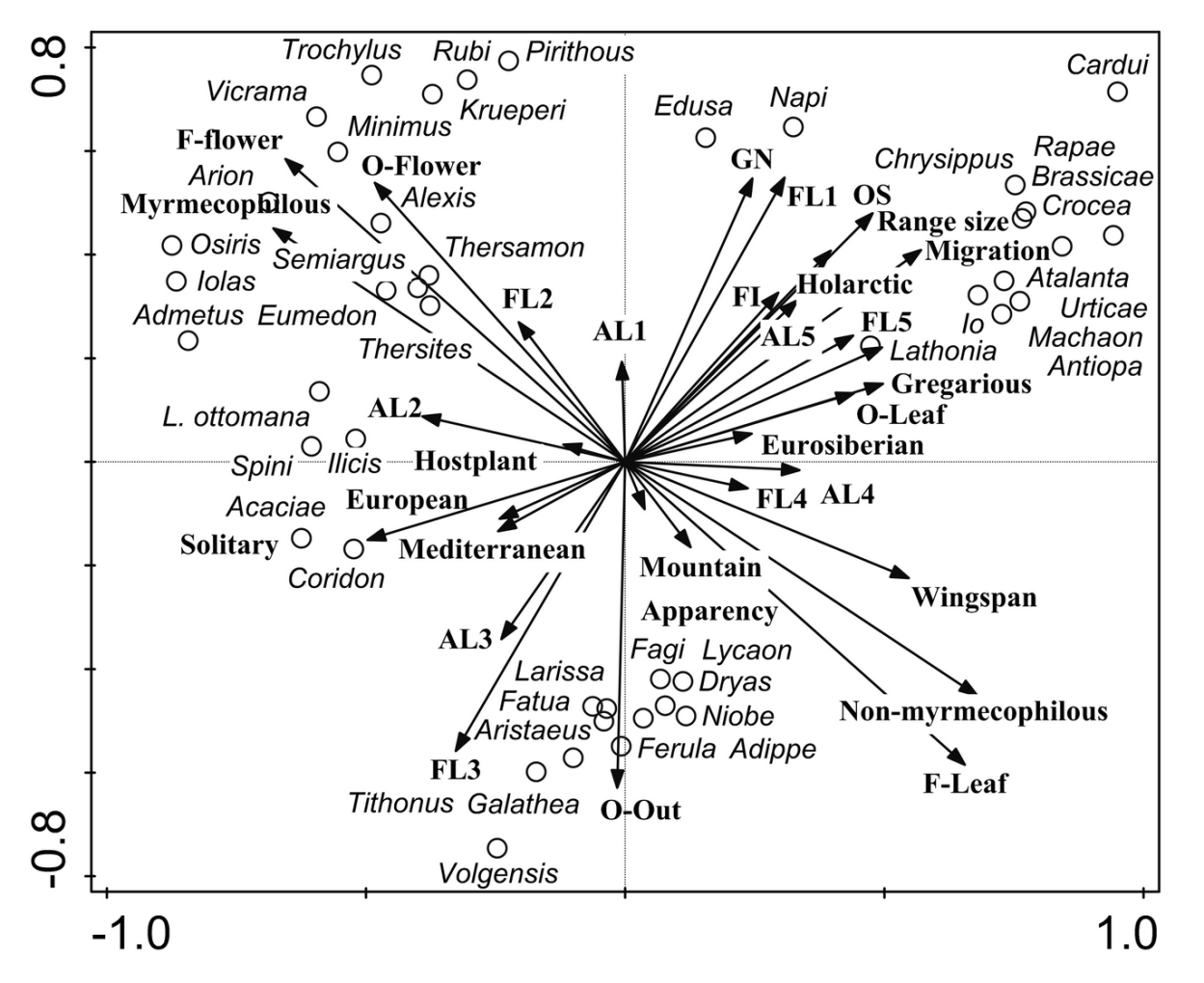


**Figure C1.** Ordination diagram of butterfly life-history traits, PCA, only species names are shown, species traits are in bold. Abbreviation as follows: AL1 (Altitudinal range): 0–500 m, AL2: 501–2000 m, AL3: over 2001; FL1 (Flying period): February – March, FL2: April–beginning of June, FL3: until the end of June, FL4: half of July–September, FL5: October and onward; F-flower: feeding on flower; F-Leaf: feeding on leaves; O-Flower: ovipositing on flowers; O-Leaf : ovipositing on hostplant leaves; O-out: ovipositing outside the hostplant; GN: Generations number; OS: overwintering stage.

**References**

1. Ter Braak CJF, Smilauer P. Canoco 5, Windows release (5.00) 2013 [cited 2014 February 5, 2014]. Available from: [www.canoco5.com](http://www.canoco5.com).
