# Supplementary material for: Life History Traits Reflect Changes in Mediterranean Butterfly Communities Due to Forest Encroachment
Source: PLoS One. 2016 Mar 21;11(3):e0152026. doi: 10.1371/journal.pone.0152026 (PMC4801352; doi:10.1371/journal.pone.0152026)
Supplement: S1 Table — (DOCX) [file pone.0152026.s004.docx]

**Life History Traits Reflect Changes in Mediterranean Butterfly Communities due to Forest Encroachment**

**Short title: Forest Encroachment and Mediterranean Butterflies**

Jana Slancarova^1,2*^, Alena Bartonova^1,2^, Michal Zapletal^1,2^, Milan Kotilinek^1^, Zdenek Faltynek Fric^2^, Nikola Micevski^3^, Vasiliki Kati^4^, , Martin Konvicka^1,2*^

^1^ Faculty of Science, University of South Bohemia, Ceske Budejovice, Czech Republic

^2^ Institute of Entomology, Biology Centre CAS, Ceske Budejovice, Czech Republic

^3^ Macedonian Entomological Society (ENTOMAK), Skopje, Republic of Macedonia (FYROM)

^4^ Department of Environmental and Natural Resources Management, University of Patras,

Agrinio, Greece

^*^ corresponding authors, emails: konva333@gmail.com (MK), slancaro@mail.com (JS)

**S1 Table. Checklist of butterfly species recorded in individual regions and total numbers of records (+/– indicate presence/absence).** Nomenclature follows De Jong [1], Red List categorization follows Van Swaay et al. [2]. Following species were not identified in the field: *Gegenes nostrodamus/pumilio, Hipparchia statilinus/fatua, Hipparchia senthes/volgensis, Leptidea sinapis/reali*, *Aricia anteros/agestis.*

| **Scientific name** | **R1** | **R2** | **R3** | **R4** | **R5** | **Presence in**  ***grassland*** | **Presence in**  ***open formations*** | **Presence in *scrub***  ***fores*t** | **Red -listed Species** |
| --- | --- | --- | --- | --- | --- | --- | --- | --- | --- |
| HESPERIIDAE |  |  |  |  |  |  |  |  |  |
| *Carcharodus alceae* (Esper, 1780) | + | + | + | + | + | 6 | 7 | 3 |  |
| *Carcharodus orientalis* Reverdin, 1913 | + | – | + | + | – | 14 | 10 | 10 |  |
| *Erynnis marloyi* (Boisduval, 1834) | – | – | + | – | – | 2 | 1 | 0 |  |
| *Erynnis tages* (Linnaeus, 1758) | + | + | – | + | + | 5 | 6 | 1 |  |
| *Gegenes nostrodamus* (Fabricius, 1793) | + | + | – | – | – | 0 | 1 | 1 |  |
| *Gegenes pumilio* (Hoffmannsegg, 1804) | + | + | – | – | – | 3 | 1 | 0 |  |
| *Muschampia proto* (Ochsenheimer, 1808) | + | + | + | – | – | 19 | 17 | 12 |  |
| *Ochlodes sylvanus* (Esper, 1777) | – | + | – | + | + | 0 | 2 | 1 |  |
| *Pyrgus armoricanus* (Oberthür, 1910) | + | – | – | + | + | 12 | 9 | 2 |  |
| *Pyrgus cinarae* (Rambur, 1839) | – | – | – | + | + | 4 | 4 | 1 |  |
| *Pyrgus malvae* (Linnaeus, 1758) | + | – | – | + | + | 5 | 2 | 2 |  |
| *Pyrgus serratulae* (Rambur, 1839) | – | – | – | + | – | 0 | 1 | 0 |  |
| *Pyrgus sidae* (Esper, 1784) | + | – | – | + | + | 6 | 10 | 3 |  |
| *Spialia orbifer* (Hübner, 1823) | + | + | + | + | + | 22 | 15 | 2 |  |
| *Thymelicus acteon* (Rottemburg, 1775) | + | + | + | + | + | 5 | 12 | 6 | + |
| *Thymelicus lineola* (Ochsenheimer, 1808) | – | – | – | + | – | 0 | 0 | 1 |  |
| *Thymelicus sylvestris* (Poda, 1761) | + | + | + | + | + | 27 | 29 | 24 |  |
| PAPILIONIDAE |  |  |  |  |  |  |  |  |  |
| *Iphiclides podalirius* (Linnaeus, 1758) | + | + | + | + | + | 38 | 37 | 27 |  |
| *Papilio alexanor* Esper, 1800 | + | + | + | – | – | 5 | 8 | 8 |  |
| *Papilio machaon* Linnaeus, 1758 | + | + | + | + | + | 13 | 10 | 9 |  |
| *Parnassius mnemosyne* (Linnaeus, 1758) | – | – | – | – | + | 1 | 2 | 1 | + |
| *Zerynthia cerisy* (Godart, 1824) | – | – | – | + | + | 3 | 1 | 2 | + |
| *Zerynthia polyxena* (Denis & Schiffermüller, 1775) | – | + | + | – | – | 3 | 2 | 1 |  |
| PIERIDAE |  |  |  |  |  |  |  |  |  |
| *Anthocharis cardamines* (Linnaeus, 1758) | + | + | + | + | + | 14 | 13 | 14 |  |
| *Anthocharis gruneri* Herrich-Schäffer, 1851 | + | + | + | – | – | 6 | 5 | 1 |  |
| **Scientific name** | **R1** | **R2** | **R3** | **R4** | **R5** | **Presence in**  ***grassland*** | **Presence in**  ***open formations*** | **Presence in *scrub***  ***forest*** | **Red List** |
| *Aporia crataegi* (Linnaeus, 1758) | + | + | + | + | + | 33 | 28 | 34 |  |
| *Colias alfacariensis* Ribbe, 1905 | – | – | – | + | – | 5 | 5 | 2 |  |
| *Colias crocea* (Geoffroy, 1785) | + | + | + | + | + | 49 | 42 | 34 |  |
| *Euchloe ausonia* (Hübner, 1804) | + | + | + | + | + | 7 | 7 | 0 |  |
| *Gonepteryx cleopatra* (Linnaeus, 1767) | + | + | + | – | – | 12 | 19 | 15 |  |
| *Gonepteryx farinosa* (Zeller, 1847) | – | + | + | – | – | 2 | 1 | 1 |  |
| *Gonepteryx rhamni* (Linnaeus, 1758) | + | + | + | + | – | 4 | 7 | 3 |  |
| *Leptidea duponcheli* (Staudinger, 1871) | – | – | + | + | – | 1 | 1 | 0 |  |
| *Leptidea sinapis* (Linnaeus, 1758) | + | + | + | + | + | 17 | 18 | 20 |  |
| *Pieris brassicae* (Linnaeus, 1758) | + | + | + | – | – | 4 | 2 | 3 |  |
| *Pieris krueperi* Staudinger, 1860 | – | – | + | – | – | 2 | 0 | 1 |  |
| *Pieris mannii* (Mayer, 1851) | + | – | – | – | + | 3 | 0 | 3 |  |
| *Pieris napi* (Linnaeus, 1758) | – | – | + | + | + | 2 | 3 | 5 |  |
| *Pieris rapae* (Linnaeus, 1758) | + | + | + | + | + | 24 | 23 | 19 |  |
| *Pontia edusa* (Fabricius, 1777) | + | + | + | + | + | 33 | 21 | 13 |  |
| LYCAENIDAE |  |  |  |  |  |  |  |  |  |
| *Aricia agestis* (Denis & Schiffermüller, 1775) | + | + | + | + | + | 40 | 40 | 28 |  |
| *Aricia anteros* (Freyer, 1838) | – | – | – | – | + | 0 | 1 | 1 | + |
| *Aricia eumedon* (Esper, 1780) | – | – | – | + | – | 0 | 0 | 1 |  |
| *Callophrys rubi* (Linnaeus, 1758) | + | + | + | + | + | 15 | 15 | 24 |  |
| *Celastrina argiolus* (Linnaeus, 1758) | + | + | + | + | + | 2 | 2 | 6 |  |
| *Chilades trochylus* (Freyer, 1845) | – | + | + | – | – | 2 | 0 | 1 |  |
| *Cupido minimus* (Fuessly, 1775) | + | – | + | + | – | 2 | 2 | 2 |  |
| *Cupido osiris* (Meigen, 1829) | + | – | – | + | – | 2 | 4 | 2 |  |
| *Cyaniris semiargus* (Rottemburg, 1775) | + | + | – | + | + | 6 | 9 | 10 |  |
| *Favonius quercus* (Linnaeus, 1758) | + | + | + | – | + | 1 | 4 | 9 |  |
| *Glaucopsyche alexis* (Poda, 1761) | + | + | – | + | + | 9 | 6 | 3 |  |
| *Iolana iolas* (Ochsenheimer, 1816) | – | – | – | + | – | 1 | 0 | 1 | + |
| *Leptotes pirithous* (Linnaeus, 1767) | + | + | + | + | – | 3 | 3 | 4 |  |
| *Lycaena alciphron* (Rottemburg, 1775) | – | – | – | + | – | 0 | 0 | 1 |  |
| *Lycaena ottomana* (Lefèbvre, 1830) | + | + | + | – | – | 2 | 4 | 3 |  |
| *Lycaena phlaeas* (Linnaeus, 1761) | + | + | + | + | + | 25 | 25 | 21 |  |
| *Lycaena thersamon* (Esper, 1784) | – | – | – | + | – | 3 | 2 | 1 |  |
| *Lycaena tityrus* (Poda, 1761) | – | – | – | + | + | 11 | 7 | 5 |  |
| *Phengaris arion* (Linnaeus, 1758) | – | – | – | + | – | 1 | 1 | 1 |  |
| *Plebejus argus* (Linnaeus, 1758) | + | + | – | + | + | 15 | 13 | 5 |  |
| *Plebejus sephirus* (Frivaldszky, 1835) | – | – | – | + | + | 2 | 3 | 0 |  |
| *Polyommatus admetus* (Esper, 1783) | – | – | + | + | – | 0 | 1 | 2 |  |
| **Scientific name** | **R1** | **R2** | **R3** | **R4** | **R5** | **Presence in**  ***grassland*** | **Presence in**  ***open formations*** | **Presence in *scrub***  ***forest*** | **Red List** |
| *Polyommatus bellargus* (Rottemburg, 1775) | + | – | – | + | – | 7 | 9 | 7 |  |
| *Polyommatus coridon* (Poda, 1761) | – | – | – | + | – | 1 | 1 | 0 |  |
| *Polyommatus dorylas* (Schiffermüller, 1775) | + | – | – | – | – | 2 | 1 | 0 | + |
| *Polyommatus icarus* (Rottemburg, 1775) | + | + | + | + | + | 48 | 47 | 40 |  |
| *Polyommatus thersites* (Cantener, 1835) | + | + | + | + | + | 19 | 24 | 15 |  |
| *Pseudophilotes vicrama* (Moore, 1865) | + | + | + | + | + | 10 | 8 | 1 | + |
| *Satyrium acaciae* (Fabricius, 1787) | – | – | – | + | + | 6 | 4 | 0 |  |
| *Satyrium ilicis* (Esper, 1779) | + | + | + | + | + | 13 | 18 | 20 |  |
| *Satyrium spini* (Denis & Schiffermüller, 1775) | + | + | + | + | – | 6 | 6 | 6 |  |
| *Satyrium w-album* (Knoch, 1782) | – | + | – | – | – | 1 | 0 | 0 | + |
| *Tarucus balkanicus* (Freyer, 1844) | + | – | – | + | – | 1 | 3 | 2 |  |
| NYMPHALIDAE |  |  |  |  |  |  |  |  |  |
| *Aglais io* (Linnaeus, 1758) | + | – | + | – | + | 3 | 1 | 1 |  |
| *Aglais urticae* (Linnaeus, 1758) | – | – | – | – | + | 1 | 0 | 1 |  |
| *Aphantopus hyperantus* (Linnaeus, 1758) | + | – | – | + | – | 1 | 1 | 1 |  |
| *Arethusana arethusa* (Denis & Schiffermüller, 1775) | – | – | – | + | + | 4 | 5 | 5 |  |
| *Argynnis adippe* (Denis & Schiffermüller, 1775) | + | – | – | + | – | 1 | 1 | 0 |  |
| *Argynnis aglaja* (Linnaeus, 1758) | – | – | – | – | + | 1 | 0 | 1 |  |
| *Argynnis niobe* (Linnaeus, 1758) | + | – | – | + | + | 5 | 6 | 5 |  |
| *Argynnis pandora* (Denis & Schiffermüller, 1775) | + | – | + | + | + | 12 | 7 | 9 |  |
| *Argynnis paphia* (Linnaeus, 1758) | + | + | + | + | + | 12 | 15 | 14 |  |
| *Boloria dia* (Linnaeus, 1767) | – | – | – | – | + | 0 | 0 | 1 |  |
| *Brenthis daphne* (Bergsträsser, 1780) | – | – | – | + | + | 5 | 4 | 2 |  |
| *Brenthis hecate* (Denis & Schiffermüller, 1775) | – | – | – | + | + | 3 | 7 | 6 |  |
| *Brintesia circe* (Fabricius, 1775) | + | + | + | + | + | 24 | 29 | 24 |  |
| *Coenonympha arcania* (Linnaeus, 1761) | – | – | – | + | – | 1 | 0 | 2 |  |
| *Coenonympha leander* (Esper, 1784) | – | – | – | + | – | 1 | 2 | 2 |  |
| *Coenonympha pamphilus* (Linnaeus, 1758) | + | + | + | + | + | 37 | 34 | 28 |  |
| *Danaus chrysippus* (Linnaeus, 1758) | – | + | – | – | – | 1 | 0 | 0 |  |
| *Erebia medusa* (Denis & Schiffermüller, 1775) | – | – | – | + | – | 1 | 0 | 3 |  |
| *Euphydryas aurinia* (Rottemburg, 1775) | – | – | – | + | + | 1 | 0 | 2 |  |
| *Hipparchia aristaeus* (Bonelli, 1826) | + | + | + | + | – | 2 | 6 | 2 |  |
| *Hipparchia fagi* (Scopoli, 1763) | + | + | – | + | – | 1 | 4 | 1 | + |
| *Hipparchia fatua* Freyer, 1844 | + | + | + | – | + | 4 | 2 | 3 |  |
| *Hipparchia statilinus* (Hufnagel, 1766) | + | + | + | + | + | 28 | 30 | 27 | + |
| *Hipparchia syriaca* (Staudinger, 1871) | + | + | – | – | + | 1 | 3 | 2 |  |
| *Hipparchia volgensis* (Mazochin-Porshnjakov, 1952) | + | – | – | + | + | 2 | 2 | 2 |  |
| *Hyponephele lupina* (Costa, 1836) | – | + | + | + | + | 4 | 3 | 7 |  |
| **Scientific name** | **R1** | **R2** | **R3** | **R4** | **R5** | **Presence in**  ***grassland*** | **Presence in**  ***open formations*** | **Presence in *scrub***  ***forest*** | **Red List** |
| *Charaxes jasius* (Linnaeus, 1767) | + | + | – | – | – | 1 | 0 | 1 |  |
| *Chazara briseis* (Linnaeus, 1764) | – | + | – | + | + | 6 | 6 | 2 | + |
| *Hyponephele lycaon* (Kühn, 1774) | – | – | – | + | + | 2 | 0 | 3 |  |
| *Issoria lathonia* (Linnaeus, 1758) | + | – | + | + | + | 20 | 19 | 14 |  |
| *Kirinia roxelana* (Cramer, 1777) | + | + | + | + | + | 6 | 8 | 10 |  |
| *Lasiommata maera* (Linnaeus, 1758) | + | + | + | + | + | 8 | 9 | 11 |  |
| *Lasiommata megera* (Linnaeus, 1767) | + | + | + | + | + | 27 | 27 | 24 |  |
| *Libythea celtis* (Laicharting, 1782) | + | + | – | + | – | 5 | 2 | 2 |  |
| *Limenitis reducta* Staudinger, 1901 | + | + | + | + | + | 13 | 19 | 19 |  |
| *Maniola jurtina* (Linnaeus, 1758) | + | + | + | + | + | 42 | 45 | 42 |  |
| *Melanargia galathea* (Linnaeus, 1758) | + | – | – | + | + | 14 | 15 | 10 |  |
| *Melanargia larissa* (Geyer, 1828) | + | + | + | + | – | 27 | 25 | 20 |  |
| *Melitaea athalia* (Rottemburg, 1775) | – | – | – | + | + | 0 | 3 | 6 |  |
| *Melitaea cinxia* (Linnaeus, 1758) | + | + | + | + | + | 18 | 19 | 16 |  |
| *Melitaea didyma* (Esper, 1778) | + | + | + | + | + | 26 | 29 | 12 |  |
| *Melitaea phoebe* (Denis & Schiffermüller, 1775) | + | + | + | + | + | 17 | 10 | 4 |  |
| *Melitaea trivia* (Denis & Schiffermüller, 1775) | – | – | + | + | + | 7 | 12 | 4 |  |
| *Minois dryas* (Scopoli, 1763) | – | – | – | – | + | 0 | 0 | 1 |  |
| *Nymphalis antiopa* (Linnaeus, 1758) | + | + | + | – | + | 4 | 5 | 7 |  |
| *Nymphalis polychloros* (Linnaeus, 1758) | + | + | + | + | + | 11 | 7 | 5 |  |
| *Pararge aegeria* (Linnaeus, 1758) | + | + | + | + | – | 1 | 3 | 8 |  |
| *Polygonia c-album* (Linnaeus, 1758) | + | + | + | + | + | 6 | 4 | 2 |  |
| *Polygonia egea* (Cramer, 1775) | – | – | + | – | – | 0 | 1 | 0 |  |
| *Pseudochazara anthelea* (Hübner, 1824) | – | – | – | + | – | 1 | 0 | 0 |  |
| *Pyronia cecilia* (Vallantin, 1894) | + | – | – | – | – | 1 | 3 | 1 |  |
| *Pyronia tithonus* (Linnaeus, 1767) | – | – | – | + | + | 3 | 3 | 0 |  |
| *Satyrus ferula* (Fabricius, 1793) | – | + | – | + | – | 1 | 1 | 1 |  |
| *Vanessa atalanta* (Linnaeus, 1758) | + | + | + | + | – | 11 | 7 | 5 |  |
| *Vanessa cardui* (Linnaeus, 1758) | + | + | + | + | + | 33 | 29 | 15 |  |

**References**

1. De Jong YSDM. Fauna Europaea version 2.6. 2013 [cited 2014 7 July]. Available from: <http://www.faunaeur.org>.

2. Van Swaay CAM, Collins S, Maes D, Lopez Munguira M, Sasic M, Settele J, et al. European Red List of Butterflies. Luxembourg: Publications Office of the European Union; 2010. 44 p.
