# Supplementary material for: Life History Traits Reflect Changes in Mediterranean Butterfly Communities Due to Forest Encroachment
Source: PLoS One. 2016 Mar 21;11(3):e0152026. doi: 10.1371/journal.pone.0152026 (PMC4801352; doi:10.1371/journal.pone.0152026)
Supplement: S2 Table — (DOCX) [file pone.0152026.s005.docx]

**Life History Traits Reflect Changes in Mediterranean Butterfly Communities due to Forest Encroachment**

**Short title: Forest Encroachment and Mediterranean Butterflies**

Jana Slancarova^1,2*^, Alena Bartonova^1,2^, Michal Zapletal^1,2^, Milan Kotilinek^1^, Zdenek Faltynek Fric^2^, Nikola Micevski^3^, Vasiliki Kati^4^, Martin Konvicka^1,2*^

^1^ Faculty of Science, University of South Bohemia, Ceske Budejovice, Czech Republic

^2^ Institute of Entomology, Biology Centre CAS, Ceske Budejovice, Czech Republic

^3^ Macedonian Entomological Society (ENTOMAK), Skopje, Republic of Macedonia (FYROM)

^4^ Department of Environmental and Natural Resources Management, University of Patras,

Agrinio, Greece

^*^ corresponding authors, emails: konva333@gmail.com (MK), slancaro@mail.com (JS)

**S2 Table. List of butterfly species life-history traits used to analyse impacts of forest encroachment on South Balkans butterflies, associated hypotheses and relevant references.**

| **Trait name** | **Scoring** | **Levels** | **Hypothesis** | **References** |
| --- | --- | --- | --- | --- |
| **Generalism vs. specialism** | |  |  |  |
| *Feeding index* | Numeric | Measured as the number of plant families (F) and genera (G) used as larval hosts using the formula: FI = (G × F^a^)^1/2^, where a = (F)/2 ×G. The index assumes higher values for any number of hosts when the hosts represent a greater number of genera and families (i.e. increased FI) | Species with wider feeding range locate more resources in a landscape and hence should be less vulnerable by habitat change. | Garcia-Barros & Romo [1]; McKinney & Lockwood [2]; Purvis et al. [3];Woodcock et al.[4] |
| *Flight period* | Fuzzy-coded factor | FL1 – early spring: February – March; FL2 – late spring: April – beginning of June; FL3 – summer: until the end of June; FL4 – late summer: half of July – September; FL5 – autumn: October and onward | Flight period mirrors resource use of individual species, increases with increasing generalism. | Bartonova et al. [5] |
| *Generations number* | Numeric |  | Species with multiple generations per year produce more progeny per capita during favorable seasons; they hence should more rapidly build larger populations, possibly with higher numbers of disperser. | Stevens et al. [6] |
| *Migration* | Binarily |  | Migratory butterflies are highly mobile, hence unlikely threatened by local habitat alternation. Data on "mobility", probably more appropriate in generalism-specialism context, were not available for many recorded species. | Barbaro & van Halder [7] |
| *Overwintering stage* | Scale 1–4 | 1 – ovum; 4 – imago (including species without overwintering diapause) | Species overwintering in later stages should appear earlier in the season than species overwintering in early stages; then, they build new generations more rapidly, produce more generations per year, may colonize novel resources, etc. | Borschig et al. [8] |
| *Wingspan* | Numeric |  | Proposed as surrogate for mobility. On the other hand, large wingspan may be due to feeding on unapparent resources, which constrain annual number of generations and selects for high fecundity. | Mobility: Sekar [9], generations number: Cizet et al. [10] |
| **Larval feeding habit** | |  |  |  |
| *Gregariousness* | Binarily |  | *Gregarious caterpillars feed more efficiently and develop faster than solitary ones* | e.g. Clark & Faeth [11] |
| *Host plant form* | Scale 1–4 | 1 – ephemers and small herbs; 2 – higher herbs and grasses; 3 – shrubs and small trees; 4 – trees | Apparent hosts tend to be large, dominating their habitats, and persistent; they are hence more easily located in a landscape. They generalized antiherbivore protection attracts species with wider trophic ranges. | Bartonova et al. [5] |
| *Larval feeding mode* | Fuzzy | Leaf-feeder; flower-feeder | Feeding on nutrient-rich flowers and developing fruits may accelerate development, allowing increase in number of generations. On the other hand, exclusive flower feeders might be confined to one generation simply because the appropriate plant parts are available only for a short time. | Cizek et al. [10] |
| *Myrmecophily* | Binarily | Myrmecophilous/Non-myrmecophilous | The mutualistic association with ants enhances juvenile survivorship and hastens development; on the other hand, it can produce risky overspecialization. | Pierce et al. , [12]; Thomas & Elmes [13] |
| *Ovum placement* | Categorical | Host plant *Flower*, host plant *Leaf*, wherever at *Hostplant*, *Out* of host plant | Specialization on particular plant parts restricts time window available for oviposition or larval development. | Cizek et al. [10] |
| **Distribution type** | |  |  |  |
| *Altitudinal range* | Fuzzy | AL1: 0–500 m a. s. l.; AL2: 501–2000 m a. s. l.; AL3: over 2001 m a. s. l. | Wide altitude range should buffer against environmental change, either human induced (e.g., intensive farming in lowlands), or even climatic. | Illan et al. [14] |
| *Mountain distribution* | Scale 0–1 | 0 – lowland presence; 0.5 – lowlands and mountains presence; 1 – only mountain presence. As for mountains were considered Alps, Pyrenees, Carpathian Mountains, Balkan mountains. | Species exclusive to mountains should be less affected by land use change in Mediterranean proper. | Roth et al. [15] |
| *Range size* | Scale 1–4 | 1 – small endemic range (including peninsulas smaller than Iberian); 2 – size of Europe or Mediterranean; 3 – Western Palearctic (Europe as far as Ural, Western Kazakhstan, Pontomediterranean range); 4 – Eurosiberian (further than Ural, including circumpolar range); 5 – huge (larger than Palearctic) | Range size should be inversely proportional to decline risk. | Brown [16]; Garcia-Barros & Romo [1] |
| *Range type* | Categorical | European; Eurosiberian; Mediterranean, Holarctic | Species with Mediterranean and/or European ranges are of higher conservation concern, as they likely include range restricted endemics. | Myers et al. [17]; Zografou et al. [18] |

**References**

1. Garcia-Barros E, Romo H. The relationship between geographic range size and life history traits: is biogeographic history uncovered? A test using the Iberian butterflies. Ecography. 2010;33:392-401. doi: 10.1111/j.1600-0587.2010.06269.x.

2. McKinney ML, Lockwood JL. Biotic homogenization: a few winners replacing many losers in the next mass extinction. Trends Ecol Evol. 1999;14:450-3.

3. Purvis A, Gittleman JL, Cowlishaw G, Mace GM. Predicting extinction risk in declining species. Proceedings of the Royal Society B-Biological Sciences. 2000;267:1947-52.

4. Woodcock BA, Bullock JM, Mortimer SR, Brereton T, Redhead JW, Thomas JA, et al. Identifying time lags in the restoration of grassland butterfly communities: A multi-site assessment. Biological Conservation. 2012;155:50-8. doi: 10.1016/j.biocon.2012.05.013.

5. Bartonova A, Benes J, Konvicka M. Generalist-specialist continuum and life history traits of Central European butterflies (Lepidoptera) - are we missing a part of the picture? European Journal of Entomology. 2014;111:543-53. doi: 10.14411/eje.2014.060.

6. Stevens VM, Trochet A, Van Dyck H, Clobert J, Baguette M. How is dispersal integrated in life histories: a quantitative analysis using butterflies. Ecol Lett. 2012;15:74-86. doi: 10.1111/j.1461-0248.2011.01709.x.

7. Barbaro L, van Halder I. Linking bird, carabid beetle and butterfly life-history traits to habitat fragmentation in mosaic landscapes. Ecography. 2009;32:321-33. doi: 10.1111/j.1600-0587.2008.05546.x.

8. Borschig C, Klein AM, von Wehrden H, Krauss J. Traits of butterfly communities change from specialist to generalist characteristics with increasing land-use intensity. Basic and Applied Ecology. 2013;14:547-54. doi: 10.1016/j.baae.2013.09.002.

9. Sekar S. A meta-analysis of the traits affecting dispersal ability in butterflies: can wingspan be used as a proxy? Journal of Animal Ecology. 2012;81:174-84. doi: 10.1111/j.1365-2656.2011.01909.x.

10. Cizek L, Fric Z, Konvicka M. Host plant defences and voltinism in European butterflies. Ecol Entomol. 2006;31:337-44. doi: 10.1111/j.1365-2311.2006.00783.x.

11. Clark BR, Faeth SH. The consequences of larval aggregation in the butterfly Chlosyne lacinia. Ecol Entomol. 1997;22:408-15.

12. Pierce NE, Braby MF, Heath A, Lohman DJ, Mathew J, Rand DB, et al. The ecology and evolution of ant association in the Lycaenidae (Lepidoptera). Annu Rev Entomol. 2002;47:733-71. doi: 10.1146/annurev.ento.47.091201.145257.

13. Thomas JA, Elmes GW. Food-plant niche selection rather than the presence of ant nests explains oviposition patterns in the myrmecophilous butterfly genus Maculinea. Proceedings of the Royal Society B-Biological Sciences. 2001;268:471-7.

14. Illan JG, Gutierrez D, Diez SB, Wilson RJ. Elevational trends in butterfly phenology: implications for species responses to climate change. Ecol Entomol. 2012;37:134-44. doi: 10.1111/j.1365-2311.2012.01345.x.

15. Roth T, Plattner M, Amrhein V. Plants, Birds and Butterflies: Short-Term Responses of Species Communities to Climate Warming Vary by Taxon and with Altitude. PLoS One. 2014;9:e82490.

16. Brown JH. On the relationship between abundance and distribution of species. American Naturalist. 1984;124:255-79. doi: 10.1086/284267.

17. Myers N, Mittermeier RA, Mittermeier CG, da Fonseca GAB, Kent J. Biodiversity hotspots for conservation priorities. Nature. 2000;403:853-8. doi: 10.1038/35002501.

18. Zografou K, Sfenthourakis S, Pullin A, Kati V. On the surrogate value of red-listed butterflies for butterflies and grasshoppers: a case study in Grammos site of Natura 2000, Greece. Journal of Insect Conservation. 2009;13:505-14. doi: 10.1007/s10841-008-9198-6.
