# Supplementary material for: Life History Traits Reflect Changes in Mediterranean Butterfly Communities Due to Forest Encroachment
Source: PLoS One. 2016 Mar 21;11(3):e0152026. doi: 10.1371/journal.pone.0152026 (PMC4801352; doi:10.1371/journal.pone.0152026)
Supplement: S3 Table — (DOCX) [file pone.0152026.s006.docx]

**Life History Traits Reflect Changes in Mediterranean Butterfly Communities due to Forest Encroachment**

**Short title: Forest Encroachment and Mediterranean Butterflies**

Jana Slancarova^1,2*^, Alena Bartonova^1,2^, Michal Zapletal^1,2^, Milan Kotilinek^1^, Zdenek Faltynek Fric^2^, Nikola Micevski^3^, Vasiliki Kati^4^, Martin Konvicka^1,2*^

^1^ Faculty of Science, University of South Bohemia, Ceske Budejovice, Czech Republic

^2^ Institute of Entomology, Biology Centre CAS, Ceske Budejovice, Czech Republic

^3^ Macedonian Entomological Society (ENTOMAK), Skopje, Republic of Macedonia (FYROM)

^4^ Department of Environmental and Natural Resources Management, University of Patras,

Agrinio, Greece

^*^ corresponding authors, emails: konva333@gmail.com (MK), slancaro@mail.com (JS)

**S3 Table. List of life history traits, used to analyse impacts of forest encroachment on South Balkans butterflies.** Nomenclature follows De Jong [1]. Abbreviation as follows: *AL1* (Altitudinal range): 0–500 m, *AL2:* 501–2000 m, *AL3*: over 2001; *FL1* (Flying period): February – March, *FL2*: April–beginning of June, *FL3*: until the end of June, *FL4*: half of July–September, *FL5*: October and onward; *LGM* – Larval feeding mode; *Range type*: EUS – Eurosiberian, EUR – European, GLOB – global, HOL – Holarctic, MED – Mediterranean.

| **Scientific name** | ***Gregariousness*** | ***Myrmecophily*** | ***AL1*** | ***AL2*** | ***AL3*** | ***Feeding index*** | ***FL1*** | ***FL2*** | ***FL3*** | ***FL4*** | ***FL5*** | ***Generation numbers*** | ***Host plant apparency*** | ***LFM – flower*** | ***LFM – leaf*** | ***Migration*** | ***Mountain distribution*** | ***Overwintering stage*** | ***Ovum placement*** | ***Range size*** | ***Range type*** | ***Wingspan*** |
| --- | --- | --- | --- | --- | --- | --- | --- | --- | --- | --- | --- | --- | --- | --- | --- | --- | --- | --- | --- | --- | --- | --- |
| HESPERIIDAE |  |  |  |  |  |  |  |  |  |  |  |  |  |  |  |  |  |  |  |  |  |  |
| *Carcharodus alceae* | 0 | 0 | 0.50 | 0.50 | 0.00 | 1.94 | 0.00 | 0.25 | 0.25 | 0.25 | 0.25 | 3 | 1 | 0.00 | 1.00 | 0 | 0.50 | 2 | leaf | 4 | EUS | 14.5 |
| *Carcharodus orientalis* | 0 | 0 | 0.50 | 0.50 | 0.00 | 1.00 | 0.00 | 0.50 | 0.50 | 0.00 | 0.00 | 2 | 1 | 0.00 | 1.00 | 0 | 1.00 | 2 | out | 1 | MED | 14.5 |
| *Erynnis marloyi* | 0 | 0 | 0.50 | 0.50 | 0.00 | 1.00 | 0.00 | 0.00 | 1.00 | 0.00 | 0.00 | 1 | 1 | 0.00 | 1.00 | 0 | 1.00 | 2 | leaf | 2 | MED | 14.5 |
| *Erynnis tages* | 0 | 0 | 0.50 | 0.50 | 0.00 | 1.41 | 0.00 | 0.33 | 0.33 | 0.33 | 0.00 | 2 | 1 | 0.00 | 1.00 | 0 | 0.50 | 2 | leaf | 4 | EUS | 13.5 |
| *Gegenes nostrodamus* | 0 | 0 | 0.50 | 0.50 | 0.00 | 1.41 | 0.00 | 0.33 | 0.33 | 0.33 | 0.00 | 3 | 2 | 0.00 | 1.00 | 0 | 0.50 | 2 | leaf | 3 | MED | 15.5 |
| *Gegenes pumilio* | 0 | 0 | 0.50 | 0.50 | 0.00 | 2.00 | 0.00 | 0.33 | 0.33 | 0.33 | 0.00 | 3 | 2 | 0.00 | 1.00 | 0 | 0.50 | 2 | leaf | 3 | MED | 14.0 |
| *Muschampia proto* | 0 | 0 | 0.50 | 0.50 | 0.00 | 1.00 | 0.00 | 0.33 | 0.33 | 0.33 | 0.00 | 1 | 1 | 0.50 | 0.50 | 0 | 0.50 | 2 | hostplant | 2 | MED | 14.5 |
| *Ochlodes sylvanus* | 0 | 0 | 0.50 | 0.50 | 0.00 | 3.70 | 0.00 | 0.00 | 0.50 | 0.50 | 0.00 | 2 | 2 | 0.00 | 1.00 | 0 | 0.50 | 2 | leaf | 4 | EUS | 15.5 |
| *Pyrgus armoricanus* | 0 | 0 | 0.50 | 0.50 | 0.00 | 1.73 | 0.00 | 0.33 | 0.33 | 0.33 | 0.00 | 2 | 1 | 0.50 | 0.50 | 0 | 0.00 | 2 | hostplant | 3 | EUR | 13.0 |
| *Pyrgus cinarae* | 0 | 0 | 0.00 | 1.00 | 0.00 | 1.00 | 0.00 | 0.00 | 1.00 | 0.00 | 0.00 | 1 | 1 | 0.00 | 1.00 | 0 | 0.00 | 2 | hostplant | 3 | MED | 15.5 |
| *Pyrgus malvae* | 0 | 0 | 0.50 | 0.50 | 0.00 | 2.00 | 0.00 | 0.33 | 0.33 | 0.33 | 0.00 | 2 | 1 | 0.00 | 1.00 | 0 | 0.50 | 3 | leaf | 4 | EUS | 12.0 |
| *Pyrgus serratulae* | 0 | 0 | 0.33 | 0.33 | 0.33 | 1.00 | 0.00 | 0.50 | 0.50 | 0.00 | 0.00 | 1 | 1 | 0.50 | 0.50 | 0 | 0.50 | 2 | leaf | 4 | EUS | 13.0 |
| *Pyrgus sidae* | 0 | 0 | 0.50 | 0.50 | 0.00 | 1.00 | 0.00 | 1.00 | 0.00 | 0.00 | 0.00 | 1 | 1 | 0.50 | 0.50 | 0 | 0.50 | 2 | flower | 4 | EUS | 15.5 |
| *Spialia orbifer* | 0 | 0 | 0.50 | 0.50 | 0.00 | 1.00 | 0.00 | 0.50 | 0.50 | 0.00 | 0.00 | 2 | 1 | 0.00 | 1.00 | 0 | 0.50 | 2 | flower | 4 | EUS | 12.5 |
| *Thymelicus acteon* | 0 | 0 | 0.50 | 0.50 | 0.00 | 1.73 | 0.00 | 0.50 | 0.50 | 0.00 | 0.00 | 1 | 2 | 0.00 | 1.00 | 0 | 0.00 | 2 | leaf | 3 | EUR | 12.0 |
| *Thymelicus lineola* | 0 | 0 | 0.33 | 0.33 | 0.33 | 3.70 | 0.00 | 0.33 | 0.33 | 0.33 | 0.00 | 1 | 2 | 0.00 | 1.00 | 0 | 0.50 | 1 | hostplant | 4 | EUS | 13.0 |
| *Thymelicus sylvestris* | 0 | 0 | 0.50 | 0.50 | 0.00 | 1.73 | 0.00 | 0.50 | 0.50 | 0.00 | 0.00 | 1 | 2 | 0.00 | 1.00 | 0 | 0.50 | 2 | hostplant | 3 | EUR | 14.0 |
| **Scientific name** | ***Gregariousness*** | ***Myrmecophily*** | ***AL1*** | ***AL2*** | ***AL3*** | ***Feeding index*** | ***FL1*** | ***FL2*** | ***FL3*** | ***FL4*** | ***FL5*** | ***Generation numbers*** | ***Host plant apparency*** | ***LFM – flower*** | ***LFM – leaf*** | ***Migration*** | ***Mountain distribution*** | ***Overwintering stage*** | ***Ovum placement*** | ***Range size*** | ***Range type*** | ***Wingspan*** |
| PAPILIONIDAE |  |  |  |  |  |  |  |  |  |  |  |  |  |  |  |  |  |  |  |  |  |  |
| *Iphiclides podalirius* | 0 | 0 | 0.50 | 0.50 | 0.00 | 1.00 | 0.25 | 0.25 | 0.25 | 0.25 | 0.00 | 2 | 3 | 0.50 | 0.50 | 0 | 0.00 | 3 | leaf | 4 | EUS | 36.0 |
| *Papilio alexanor* | 0 | 0 | 0.50 | 0.50 | 0.00 | 2.83 | 0.00 | 0.50 | 0.50 | 0.00 | 0.00 | 1 | 1 | 1.00 | 0.00 | 0 | 0.00 | 3 | hostplant | 4 | MED | 32.0 |
| *Papilio machaon* | 0 | 0 | 0.33 | 0.33 | 0.33 | 2.00 | 0.20 | 0.20 | 0.20 | 0.20 | 0.20 | 2 | 1 | 0.00 | 1.00 | 0 | 0.50 | 3 | leaf | 5 | HOL | 35.0 |
| *Parnassius mnemosyne* | 0 | 0 | 0.33 | 0.33 | 0.33 | 1.00 | 0.00 | 0.33 | 0.33 | 0.33 | 0.00 | 1 | 1 | 0.00 | 1.00 | 0 | 0.50 | 1 | out | 4 | EUS | 28.5 |
| *Zerynthia cerisy* | 1 | 0 | 0.50 | 0.50 | 0.00 | 1.00 | 0.50 | 0.50 | 0.00 | 0.00 | 0.00 | 1 | 1 | 0.00 | 1.00 | 0 | 0.00 | 3 | leaf | 2 | MED | 28.5 |
| *Zerynthia polyxena* | 1 | 0 | 0.50 | 0.50 | 0.00 | 1.00 | 0.33 | 0.33 | 0.33 | 0.00 | 0.00 | 1 | 1 | 0.00 | 1.00 | 0 | 0.00 | 3 | leaf | 2 | EUR | 25.0 |
| PIERIDAE |  |  |  |  |  |  |  |  |  |  |  |  |  |  |  |  |  |  |  |  |  |  |
| *Anthocharis cardamines* | 0 | 0 | 0.50 | 0.50 | 0.00 | 2.45 | 0.50 | 0.50 | 0.00 | 0.00 | 0.00 | 1 | 1 | 0.50 | 0.50 | 0 | 0.50 | 3 | flower | 4 | EUS | 21.5 |
| *Anthocharis gruneri* | 0 | 0 | 0.50 | 0.50 | 0.00 | 1.00 | 0.50 | 0.50 | 0.00 | 0.00 | 0.00 | 1 | 1 | 0.50 | 0.50 | 0 | 0.50 | 3 | flower | 2 | MED | 17.0 |
| *Aporia crataegi* | 1 | 0 | 0.50 | 0.50 | 0.00 | 2.24 | 0.00 | 0.50 | 0.50 | 0.00 | 0.00 | 1 | 3 | 0.00 | 1.00 | 0 | 0.50 | 2 | leaf | 4 | EUS | 31.0 |
| *Colias alfacariensis* | 0 | 0 | 0.50 | 0.50 | 0.00 | 1.41 | 0.00 | 0.25 | 0.25 | 0.25 | 0.25 | 2 | 1 | 0.00 | 1.00 | 1 | 0.50 | 2 | leaf | 3 | EUR | 24.0 |
| *Colias crocea* | 0 | 0 | 0.33 | 0.33 | 0.33 | 3.87 | 0.20 | 0.20 | 0.20 | 0.20 | 0.20 | 3 | 1 | 0.00 | 1.00 | 1 | 0.50 | 4 | leaf | 3 | EUS | 25.0 |
| *Euchloe ausonia* | 0 | 0 | 0.50 | 0.50 | 0.00 | 2.65 | 0.33 | 0.33 | 0.33 | 0.00 | 0.00 | 2 | 1 | 0.50 | 0.50 | 0 | 0.00 | 3 | flower | 4 | EUS | 22.0 |
| *Gonepteryx cleopatra* | 0 | 0 | 0.33 | 0.33 | 0.33 | 1.00 | 0.33 | 0.33 | 0.33 | 0.00 | 0.00 | 1 | 3 | 0.00 | 1.00 | 0 | 0.00 | 4 | hostplant | 2 | MED | 27.5 |
| *Gonepteryx farinosa* | 0 | 0 | 0.50 | 0.50 | 0.00 | 1.41 | 0.33 | 0.33 | 0.33 | 0.00 | 0.00 | 1 | 3 | 0.00 | 1.00 | 0 | 0.00 | 4 | leaf | 3 | MED | 30.0 |
| *Gonepteryx rhamni* | 0 | 0 | 0.33 | 0.33 | 0.33 | 1.41 | 0.20 | 0.20 | 0.20 | 0.20 | 0.20 | 1 | 3 | 0.00 | 1.00 | 0 | 0.50 | 4 | hostplant | 4 | EUS | 28.0 |
| *Leptidea duponcheli* | 0 | 0 | 0.50 | 0.50 | 0.00 | 1.41 | 0.00 | 0.50 | 0.50 | 0.00 | 0.00 | 2 | 1 | 0.00 | 1.00 | 0 | 0.00 | 3 | leaf | 3 | MED | 19.0 |
| *Leptidea sinapis* | 0 | 0 | 0.33 | 0.33 | 0.33 | 1.00 | 0.00 | 0.33 | 0.33 | 0.33 | 0.00 | 2 | 1 | 0.00 | 1.00 | 0 | 0.50 | 3 | leaf | 3 | EUS | 21.5 |
| *Pieris brassicae* | 1 | 0 | 0.33 | 0.33 | 0.33 | 3.84 | 0.20 | 0.20 | 0.20 | 0.20 | 0.20 | 3 | 1 | 0.00 | 1.00 | 1 | 0.50 | 3 | leaf | 4 | EUS | 30.5 |
| *Pieris krueperi* | 0 | 0 | 0.50 | 0.50 | 0.00 | 1.00 | 0.33 | 0.33 | 0.33 | 0.00 | 0.00 | 3 | 1 | 1.00 | 0.00 | 0 | 0.00 | 3 | flower | 3 | MED | 23.0 |
| *Pieris mannii* | 0 | 0 | 0.50 | 0.50 | 0.00 | 1.41 | 0.33 | 0.33 | 0.33 | 0.00 | 0.00 | 3 | 1 | 0.00 | 1.00 | 0 | 0.50 | 3 | leaf | 2 | MED | 22.0 |
| **Scientific name** | ***Gregariousness*** | ***Myrmecophily*** | ***AL1*** | ***AL2*** | ***AL3*** | ***Feeding index*** | ***FL1*** | ***FL2*** | ***FL3*** | ***FL4*** | ***FL5*** | ***Generation numbers*** | ***Host plant apparency*** | ***LFM – flower*** | ***LFM – leaf*** | ***Migration*** | ***Mountain distribution*** | ***Overwintering stage*** | ***Ovum placement*** | ***Range size*** | ***Range type*** | ***Wingspan*** |
| *Pieris napi* | 0 | 0 | 0.50 | 0.50 | 0.00 | 3.32 | 0.25 | 0.25 | 0.25 | 0.25 | 0.00 | 3 | 1 | 0.50 | 0.50 | 1 | 0.50 | 3 | leaf | 5 | HOL | 20.0 |
| *Pieris rapae* | 0 | 0 | 0.33 | 0.33 | 0.33 | 4.85 | 0.20 | 0.20 | 0.20 | 0.20 | 0.20 | 3 | 1 | 0.00 | 1.00 | 1 | 0.50 | 3 | leaf | 4 | EUS | 25.0 |
| *Pontia edusa* | 0 | 0 | 0.33 | 0.33 | 0.33 | 2.18 | 0.25 | 0.25 | 0.25 | 0.25 | 0.00 | 3 | 1 | 1.00 | 0.00 | 1 | 0.50 | 3 | leaf | 4 | EUS | 22.5 |
| LYCAENIDAE |  |  |  |  |  |  |  |  |  |  |  |  |  |  |  |  |  |  |  |  |  |  |
| *Aricia agestis* | 0 | 1 | 0.50 | 0.50 | 0.00 | 1.94 | 0.00 | 0.33 | 0.33 | 0.33 | 0.00 | 2 | 1 | 0.00 | 1.00 | 0 | 0.50 | 2 | leaf | 4 | EUS | 13.0 |
| *Aricia anteros* | 0 | 1 | 0.00 | 1.00 | 0.00 | 1.00 | 0.00 | 0.50 | 0.50 | 0.00 | 0.00 | 2 | 1 | 0.00 | 1.00 | 0 | 1.00 | 3 | leaf | 2 | MED | 15.5 |
| *Aricia eumedon* | 0 | 1 | 0.33 | 0.33 | 0.33 | 1.00 | 0.00 | 0.50 | 0.50 | 0.00 | 0.00 | 1 | 1 | 0.50 | 0.50 | 0 | 0.50 | 2 | flower | 4 | EUS | 15.0 |
| *Callophrys rubi* | 0 | 1 | 0.50 | 0.50 | 0.00 | 4.25 | 0.50 | 0.50 | 0.00 | 0.00 | 0.00 | 1 | 3 | 0.50 | 0.50 | 0 | 0.50 | 3 | flower | 4 | EUS | 14.0 |
| *Celastrina argiolus* | 0 | 1 | 0.50 | 0.50 | 0.00 | 7.45 | 0.50 | 0.00 | 0.50 | 0.00 | 0.00 | 2 | 3 | 0.50 | 0.50 | 0 | 0.50 | 3 | flower | 5 | HOL | 15.0 |
| *Chilades trochylus* | 0 | 1 | 0.50 | 0.50 | 0.00 | 1.68 | 0.25 | 0.25 | 0.25 | 0.25 | 0.00 | 3 | 1 | 0.50 | 0.50 | 0 | 0.50 | 3 | flower | 3 | MED | 8.5 |
| *Cupido minimus* | 0 | 1 | 0.33 | 0.33 | 0.33 | 1.00 | 0.00 | 0.33 | 0.33 | 0.33 | 0.00 | 2 | 1 | 1.00 | 0.00 | 0 | 0.50 | 2 | flower | 4 | EUS | 11.0 |
| *Cupido osiris* | 0 | 1 | 0.00 | 1.00 | 0.00 | 1.00 | 0.00 | 0.50 | 0.50 | 0.00 | 0.00 | 2 | 1 | 1.00 | 0.00 | 0 | 0.50 | 2 | flower | 3 | MED | 13.5 |
| *Cyaniris semiargus* | 0 | 1 | 0.33 | 0.33 | 0.33 | 1.00 | 0.00 | 0.33 | 0.33 | 0.33 | 0.00 | 1 | 1 | 0.50 | 0.50 | 0 | 0.50 | 2 | flower | 4 | EUS | 15.5 |
| *Favonius quercus* | 0 | 1 | 0.50 | 0.50 | 0.00 | 1.00 | 0.00 | 0.00 | 0.50 | 0.50 | 0.00 | 1 | 4 | 0.50 | 0.50 | 0 | 0.50 | 1 | leaf | 3 | EUR | 13.0 |
| *Glaucopsyche alexis* | 0 | 1 | 0.50 | 0.50 | 0.00 | 3.16 | 0.00 | 0.50 | 0.50 | 0.00 | 0.00 | 1 | 1 | 0.50 | 0.50 | 0 | 0.00 | 3 | flower | 4 | EUS | 15.5 |
| *Iolana iolas* | 0 | 1 | 0.00 | 1.00 | 0.00 | 1.00 | 0.00 | 0.50 | 0.50 | 0.00 | 0.00 | 1 | 1 | 1.00 | 0.00 | 0 | 0.00 | 3 | flower | 2 | EUR | 19.5 |
| *Leptotes pirithous* | 0 | 1 | 0.50 | 0.50 | 0.00 | 4.38 | 0.25 | 0.25 | 0.25 | 0.25 | 0.00 | 3 | 2 | 0.50 | 0.50 | 1 | 0.00 | 2 | hostplant | 4 | MED | 12.5 |
| *Lycaena alciphron* | 0 | 0 | 0.33 | 0.33 | 0.33 | 1.00 | 0.00 | 0.00 | 1.00 | 0.00 | 0.00 | 1 | 1 | 0.00 | 1.00 | 0 | 0.00 | 2 | leaf | 4 | EUS | 17.0 |
| *Lycaena ottomana* | 0 | 1 | 0.50 | 0.50 | 0.00 | 1.00 | 0.00 | 0.50 | 0.50 | 0.00 | 0.00 | 2 | 1 | 0.50 | 0.50 | 0 | 1.00 | 2 | hostplant | 2 | MED | 14.5 |
| *Lycaena phlaeas* | 0 | 0 | 0.33 | 0.33 | 0.33 | 1.41 | 0.00 | 0.33 | 0.33 | 0.33 | 0.00 | 3 | 1 | 0.00 | 1.00 | 0 | 0.50 | 2 | leaf | 5 | HOL | 13.5 |
| *Lycaena thersamon* | 0 | 1 | 0.50 | 0.50 | 0.00 | 1.00 | 0.00 | 0.33 | 0.33 | 0.33 | 0.00 | 3 | 1 | 0.50 | 0.50 | 0 | 0.00 | 2 | hostplant | 4 | EUS | 15.0 |
| *Lycaena tityrus* | 0 | 0 | 0.33 | 0.33 | 0.33 | 1.00 | 0.00 | 0.25 | 0.25 | 0.25 | 0.25 | 2 | 1 | 0.00 | 1.00 | 0 | 0.50 | 2 | leaf | 4 | EUS | 15.5 |
| **Scientific name** | ***Gregariousness*** | ***Myrmecophily*** | ***AL1*** | ***AL2*** | ***AL3*** | ***Feeding index*** | ***FL1*** | ***FL2*** | ***FL3*** | ***FL4*** | ***FL5*** | ***Generation numbers*** | ***Host plant apparency*** | ***LFM – flower*** | ***LFM – leaf*** | ***Migration*** | ***Mountain distribution*** | ***Overwintering stage*** | ***Ovum placement*** | ***Range size*** | ***Range type*** | ***Wingspan*** |
| *Phengaris arion* | 0 | 1 | 0.50 | 0.50 | 0.00 | 1.00 | 0.00 | 0.50 | 0.50 | 0.00 | 0.00 | 1 | 1 | 1.00 | 0.00 | 0 | 0.50 | 2 | flower | 4 | EUS | 18.0 |
| *Plebejus argus* | 0 | 1 | 0.33 | 0.33 | 0.33 | 3.97 | 0.00 | 0.33 | 0.33 | 0.33 | 0.00 | 2 | 1 | 0.50 | 0.50 | 0 | 0.00 | 1 | out | 4 | EUS | 13.5 |
| *Plebejus sephirus* | 0 | 1 | 0.00 | 0.50 | 0.50 | 1.00 | 0.00 | 0.50 | 0.50 | 0.00 | 0.00 | 1 | 1 | 0.00 | 1.00 | 0 | 1.00 | 2 | leaf | 2 | MED | 15.5 |
| *Polyommatus admetus* | 0 | 1 | 0.50 | 0.50 | 0.00 | 1.00 | 0.00 | 0.00 | 1.00 | 0.00 | 0.00 | 1 | 1 | 1.00 | 0.00 | 0 | 0.00 | 2 | flower | 2 | MED | 17.5 |
| *Polyommatus bellargus* | 0 | 1 | 0.50 | 0.50 | 0.00 | 1.41 | 0.00 | 0.50 | 0.00 | 0.50 | 0.00 | 2 | 1 | 0.50 | 0.50 | 0 | 0.50 | 2 | leaf | 3 | EUR | 15.5 |
| *Polyommatus coridon* | 0 | 1 | 0.50 | 0.50 | 0.00 | 1.00 | 0.00 | 0.00 | 0.50 | 0.50 | 0.00 | 1 | 1 | 0.50 | 0.50 | 0 | 0.50 | 1 | out | 3 | EUR | 16.5 |
| *Polyommatus dorylas* | 0 | 1 | 0.33 | 0.33 | 0.33 | 1.00 | 0.00 | 0.50 | 0.50 | 0.00 | 0.00 | 2 | 1 | 0.50 | 0.50 | 0 | 0.50 | 2 | leaf | 3 | EUR | 16.0 |
| *Polyommatus icarus* | 0 | 1 | 0.33 | 0.33 | 0.33 | 3.46 | 0.00 | 0.25 | 0.25 | 0.25 | 0.25 | 2 | 1 | 0.50 | 0.50 | 0 | 0.50 | 2 | flower | 4 | EUS | 16.0 |
| *Polyommatus thersites* | 0 | 1 | 0.50 | 0.50 | 0.00 | 1.00 | 0.00 | 0.50 | 0.50 | 0.00 | 0.00 | 2 | 1 | 0.50 | 0.50 | 0 | 0.00 | 2 | leaf | 4 | EUS | 14.5 |
| *Pseudophilotes vicrama* | 0 | 1 | 0.50 | 0.50 | 0.00 | 1.41 | 0.00 | 0.33 | 0.33 | 0.33 | 0.00 | 2 | 1 | 1.00 | 0.00 | 0 | 0.50 | 3 | flower | 4 | EUS | 11.0 |
| *Satyrium acaciae* | 0 | 1 | 0.50 | 0.50 | 0.00 | 1.00 | 0.00 | 0.00 | 1.00 | 0.00 | 0.00 | 1 | 3 | 0.50 | 0.50 | 0 | 0.50 | 1 | hostplant | 3 | EUR | 15.0 |
| *Satyrium ilicis* | 0 | 1 | 0.50 | 0.50 | 0.00 | 1.00 | 0.00 | 0.50 | 0.50 | 0.00 | 0.00 | 1 | 3 | 0.50 | 0.50 | 0 | 0.00 | 1 | leaf | 3 | EUR | 17.0 |
| *Satyrium spini* | 0 | 1 | 0.50 | 0.50 | 0.00 | 1.41 | 0.00 | 0.50 | 0.50 | 0.00 | 0.00 | 1 | 3 | 0.50 | 0.50 | 0 | 0.50 | 1 | hostplant | 3 | EUR | 15.0 |
| *Satyrium w-album* | 0 | 1 | 0.50 | 0.50 | 0.00 | 1.00 | 0.00 | 0.00 | 1.00 | 0.00 | 0.00 | 1 | 4 | 0.50 | 0.50 | 0 | 0.00 | 1 | leaf | 4 | EUS | 15.5 |
| *Tarucus balkanicus* | 0 | 1 | 0.50 | 0.50 | 0.00 | 1.41 | 0.00 | 0.33 | 0.33 | 0.33 | 0.00 | 3 | 2 | 0.00 | 1.00 | 0 | 0.00 | 3 | hostplant | 4 | MED | 10.0 |
| NYMPHALIDAE |  |  |  |  |  |  |  |  |  |  |  |  |  |  |  |  |  |  |  |  |  |  |
| *Aglais io* | 1 | 0 | 0.33 | 0.33 | 0.33 | 1.41 | 0.25 | 0.25 | 0.25 | 0.25 | 0.00 | 2 | 2 | 0.00 | 1.00 | 1 | 0.50 | 4 | leaf | 4 | EUS | 28.0 |
| *Aglais urticae* | 1 | 0 | 0.33 | 0.33 | 0.33 | 1.00 | 0.20 | 0.20 | 0.20 | 0.20 | 0.20 | 2 | 2 | 0.00 | 1.00 | 1 | 0.50 | 4 | leaf | 4 | EUS | 23.5 |
| *Aphantopus hyperantus* | 0 | 0 | 0.50 | 0.50 | 0.00 | 4.09 | 0.00 | 0.00 | 0.50 | 0.50 | 0.00 | 1 | 2 | 0.00 | 1.00 | 0 | 0.00 | 2 | out | 4 | EUS | 22.0 |
| *Arethusana arethusa* | 0 | 0 | 0.50 | 0.50 | 0.00 | 2.24 | 0.00 | 0.00 | 0.50 | 0.50 | 0.00 | 1 | 2 | 0.00 | 1.00 | 0 | 0.50 | 2 | out | 4 | EUS | 23.0 |
| *Argynnis adippe* | 0 | 0 | 0.50 | 0.50 | 0.00 | 1.00 | 0.00 | 0.00 | 0.50 | 0.50 | 0.00 | 1 | 1 | 0.00 | 1.00 | 0 | 0.50 | 1 | out | 4 | EUS | 28.0 |
| *Argynnis aglaja* | 0 | 0 | 0.33 | 0.33 | 0.33 | 1.00 | 0.00 | 0.00 | 1.00 | 0.00 | 0.00 | 1 | 1 | 0.00 | 1.00 | 0 | 0.50 | 2 | leaf | 4 | EUS | 28.0 |
| *Argynnis niobe* | 0 | 0 | 0.33 | 0.33 | 0.33 | 1.00 | 0.00 | 0.00 | 0.50 | 0.50 | 0.00 | 1 | 1 | 0.00 | 1.00 | 0 | 0.50 | 1 | out | 4 | EUS | 26.5 |
| **Scientific name** | ***Gregariousness*** | ***Myrmecophily*** | ***AL1*** | ***AL2*** | ***AL3*** | ***Feeding index*** | ***FL1*** | ***FL2*** | ***FL3*** | ***FL4*** | ***FL5*** | ***Generation numbers*** | ***Host plant apparency*** | ***LFM – flower*** | ***LFM – leaf*** | ***Migration*** | ***Mountain distribution*** | ***Overwintering stage*** | ***Ovum placement*** | ***Range size*** | ***Range type*** | ***Wingspan*** |
| *Argynnis pandora* | 0 | 0 | 0.50 | 0.50 | 0.00 | 1.00 | 0.00 | 0.50 | 0.50 | 0.00 | 0.00 | 1 | 1 | 0.00 | 1.00 | 0 | 0.00 | 2 | out | 4 | EUS | 36.0 |
| *Argynnis paphia* | 0 | 0 | 0.50 | 0.50 | 0.00 | 1.00 | 0.00 | 0.00 | 0.50 | 0.50 | 0.00 | 1 | 1 | 0.00 | 1.00 | 0 | 0.00 | 2 | hostplant | 4 | EUS | 31.0 |
| *Boloria dia* | 0 | 0 | 0.00 | 1.00 | 0.00 | 1.00 | 0.00 | 0.33 | 0.33 | 0.33 | 0.00 | 2 | 1 | 0.00 | 1.00 | 0 | 0.50 | 2 | leaf | 4 | EUS | 16.5 |
| *Brenthis daphne* | 0 | 0 | 0.50 | 0.50 | 0.00 | 1.00 | 0.00 | 0.00 | 1.00 | 0.00 | 0.00 | 1 | 3 | 0.00 | 1.00 | 0 | 0.50 | 1 | leaf | 4 | EUS | 23.5 |
| *Brenthis hecate* | 0 | 0 | 0.50 | 0.50 | 0.00 | 1.00 | 0.00 | 0.00 | 1.00 | 0.00 | 0.00 | 1 | 1 | 0.00 | 1.00 | 0 | 0.50 | 1 | leaf | 4 | EUS | 20.0 |
| *Brintesia circe* | 0 | 0 | 0.50 | 0.50 | 0.00 | 1.41 | 0.00 | 0.00 | 0.50 | 0.50 | 0.00 | 1 | 2 | 0.00 | 1.00 | 0 | 0.00 | 2 | out | 4 | EUS | 34.5 |
| *Charaxes jasius* | 0 | 0 | 0.33 | 0.33 | 0.33 | 1.00 | 0.00 | 0.33 | 0.33 | 0.33 | 0.00 | 2 | 2 | 0.00 | 1.00 | 0 | 0.00 | 2 | leaf | 2 | MED | 39.5 |
| *Chazara briseis* | 0 | 0 | 0.50 | 0.50 | 0.00 | 1.41 | 0.00 | 0.00 | 0.33 | 0.33 | 0.33 | 1 | 2 | 0.00 | 1.00 | 0 | 0.50 | 2 | out | 4 | EUS | 25.5 |
| *Coenonympha arcania* | 0 | 0 | 0.50 | 0.50 | 0.00 | 1.73 | 0.00 | 0.00 | 0.50 | 0.50 | 0.00 | 1 | 2 | 0.00 | 1.00 | 0 | 0.50 | 2 | leaf | 3 | EUR | 18.5 |
| *Coenonympha leander* | 0 | 0 | 0.50 | 0.50 | 0.00 | 1.41 | 0.00 | 0.50 | 0.50 | 0.00 | 0.00 | 1 | 2 | 0.00 | 1.00 | 0 | 1.00 | 2 | out | 3 | MED | 16.5 |
| *Coenonympha pamphilus* | 0 | 0 | 0.50 | 0.50 | 0.00 | 2.45 | 0.20 | 0.20 | 0.20 | 0.20 | 0.20 | 3 | 2 | 0.00 | 1.00 | 0 | 0.50 | 2 | out | 4 | EUS | 15.0 |
| *Danaus chrysippus* | 1 | 0 | 0.50 | 0.50 | 0.00 | 1.41 | 0.20 | 0.20 | 0.20 | 0.20 | 0.20 | 3 | 2 | 0.00 | 1.00 | 1 | 0.00 | 4 | leaf | 5 | MED | 38.0 |
| *Erebia medusa* | 0 | 0 | 0.33 | 0.33 | 0.33 | 1.73 | 0.00 | 0.50 | 0.50 | 0.00 | 0.00 | 1 | 2 | 0.00 | 1.00 | 0 | 0.50 | 2 | hostplant | 4 | EUS | 22.5 |
| *Euphydryas aurinia* | 1 | 0 | 0.33 | 0.33 | 0.33 | 3.09 | 0.00 | 0.50 | 0.50 | 0.00 | 0.00 | 1 | 2 | 0.00 | 1.00 | 0 | 0.50 | 2 | leaf | 4 | EUS | 18.0 |
| *Hipparchia aristaeus* | 0 | 0 | 0.33 | 0.33 | 0.33 | 1.73 | 0.00 | 0.50 | 0.50 | 0.00 | 0.00 | 1 | 2 | 0.00 | 1.00 | 0 | 0.50 | 2 | out | 2 | MED | 26.0 |
| *Hipparchia fagi* | 0 | 0 | 0.50 | 0.50 | 0.00 | 1.73 | 0.00 | 0.00 | 0.50 | 0.50 | 0.00 | 1 | 2 | 0.00 | 1.00 | 0 | 0.00 | 2 | out | 3 | EUS | 30.0 |
| *Hipparchia fatua* | 0 | 0 | 0.50 | 0.50 | 0.00 | 1.41 | 0.00 | 0.33 | 0.33 | 0.33 | 0.00 | 1 | 2 | 0.00 | 1.00 | 0 | 0.00 | 2 | out | 2 | MED | 29.0 |
| *Hipparchia statilinu* | 0 | 0 | 0.33 | 0.33 | 0.33 | 2.00 | 0.00 | 0.00 | 0.50 | 0.50 | 0.00 | 1 | 2 | 0.00 | 1.00 | 0 | 0.50 | 2 | out | 3 | EUR | 22.5 |
| *Hipparchia syriaca* | 0 | 0 | 0.50 | 0.50 | 0.00 | 1.41 | 0.00 | 0.50 | 0.50 | 0.00 | 0.00 | 1 | 2 | 0.00 | 1.00 | 0 | 0.00 | 2 | out | 3 | MED | 35.5 |
| *Hipparchia volgensis* | 0 | 0 | 0.00 | 1.00 | 0.00 | 1.00 | 0.00 | 0.00 | 1.00 | 0.00 | 0.00 | 1 | 2 | 0.00 | 1.00 | 0 | 0.50 | 2 | out | 2 | MED | 23.5 |
| *Hyponephele lupina* | 0 | 0 | 0.33 | 0.33 | 0.33 | 2.24 | 0.00 | 0.50 | 0.50 | 0.00 | 0.00 | 1 | 2 | 0.00 | 1.00 | 0 | 0.50 | 2 | out | 4 | EUS | 23.5 |
| *Hyponephele lycaon* | 0 | 0 | 0.50 | 0.50 | 0.00 | 1.73 | 0.00 | 0.00 | 0.50 | 0.50 | 0.00 | 1 | 2 | 0.00 | 1.00 | 0 | 0.50 | 2 | out | 4 | EUS | 22.0 |
| **Scientific name** | ***Gregariousness*** | ***Myrmecophily*** | ***AL1*** | ***AL2*** | ***AL3*** | ***Feeding index*** | ***FL1*** | ***FL2*** | ***FL3*** | ***FL4*** | ***FL5*** | ***Generation numbers*** | ***Host plant apparency*** | ***LFM – flower*** | ***LFM – leaf*** | ***Migration*** | ***Mountain distribution*** | ***Overwintering stage*** | ***Ovum placement*** | ***Range size*** | ***Range type*** | ***Wingspan*** |
| *Issoria lathonia* | 0 | 0 | 0.33 | 0.33 | 0.33 | 1.00 | 0.20 | 0.20 | 0.20 | 0.20 | 0.20 | 3 | 1 | 0.00 | 1.00 | 1 | 0.50 | 2 | leaf | 4 | EUS | 21.0 |
| *Kirinia roxelana* | 0 | 0 | 0.50 | 0.50 | 0.00 | 2.00 | 0.00 | 0.50 | 0.50 | 0.00 | 0.00 | 1 | 2 | 0.00 | 1.00 | 0 | 0.00 | 2 | hostplant | 2 | MED | 30.0 |
| *Lasiommata maera* | 0 | 0 | 0.50 | 0.50 | 0.00 | 3.12 | 0.00 | 0.33 | 0.33 | 0.33 | 0.00 | 2 | 2 | 0.00 | 1.00 | 0 | 0.50 | 2 | hostplant | 4 | EUS | 26.5 |
| *Lasiommata megera* | 0 | 0 | 0.33 | 0.33 | 0.33 | 2.45 | 0.00 | 0.25 | 0.25 | 0.25 | 0.25 | 2 | 2 | 0.00 | 1.00 | 0 | 0.50 | 3 | outout | 3 | EUR | 22.0 |
| *Libythea celtis* | 0 | 0 | 0.50 | 0.50 | 0.00 | 1.00 | 0.00 | 0.50 | 0.50 | 0.00 | 0.00 | 1 | 4 | 0.00 | 1.00 | 1 | 0.50 | 4 | leaf | 4 | EUS | 19.5 |
| *Limenitis reducta* | 0 | 0 | 0.50 | 0.50 | 0.00 | 1.00 | 0.00 | 0.50 | 0.50 | 0.00 | 0.00 | 2 | 3 | 0.00 | 1.00 | 0 | 0.00 | 2 | leaf | 2 | EUR | 25.0 |
| *Maniola jurtina* | 0 | 0 | 0.50 | 0.50 | 0.00 | 3.16 | 0.00 | 0.00 | 0.50 | 0.50 | 0.00 | 1 | 2 | 0.00 | 1.00 | 0 | 0.00 | 2 | out | 3 | EUS | 23.5 |
| *Melanargia galathea* | 0 | 0 | 0.50 | 0.50 | 0.00 | 3.00 | 0.00 | 0.00 | 1.00 | 0.00 | 0.00 | 1 | 2 | 0.00 | 1.00 | 0 | 0.50 | 2 | out | 3 | EUR | 24.5 |
| *Melanargia larissa* | 0 | 0 | 0.33 | 0.33 | 0.33 | 1.00 | 0.00 | 0.50 | 0.50 | 0.00 | 0.00 | 1 | 2 | 0.00 | 1.00 | 0 | 0.50 | 2 | out | 2 | MED | 27.5 |
| *Melitaea athalia* | 1 | 0 | 0.33 | 0.33 | 0.33 | 2.40 | 0.00 | 0.33 | 0.33 | 0.33 | 0.00 | 1 | 1 | 0.00 | 1.00 | 0 | 0.50 | 2 | leaf | 4 | EUS | 19.0 |
| *Melitaea cinxia* | 1 | 0 | 0.50 | 0.50 | 0.00 | 2.46 | 0.00 | 0.33 | 0.33 | 0.33 | 0.00 | 2 | 1 | 0.00 | 1.00 | 0 | 0.50 | 2 | leaf | 4 | EUS | 18.0 |
| *Melitaea didyma* | 1 | 0 | 0.33 | 0.33 | 0.33 | 3.50 | 0.00 | 0.33 | 0.33 | 0.33 | 0.00 | 2 | 1 | 0.00 | 1.00 | 0 | 0.50 | 2 | leaf | 4 | EUS | 20.0 |
| *Melitaea phoebe* | 1 | 0 | 0.50 | 0.50 | 0.00 | 1.41 | 0.00 | 0.33 | 0.33 | 0.33 | 0.00 | 2 | 1 | 0.00 | 1.00 | 0 | 0.50 | 2 | leaf | 4 | EUS | 22.0 |
| *Melitaea trivia* | 1 | 0 | 0.50 | 0.50 | 0.00 | 1.00 | 0.00 | 0.50 | 0.50 | 0.00 | 0.00 | 2 | 1 | 0.00 | 1.00 | 0 | 0.00 | 2 | leaf | 4 | EUS | 18.0 |
| *Minois dryas* | 0 | 0 | 0.50 | 0.50 | 0.00 | 2.24 | 0.00 | 0.00 | 0.50 | 0.50 | 0.00 | 1 | 2 | 0.00 | 1.00 | 0 | 0.50 | 2 | out | 4 | EUS | 28.0 |
| *Nymphalis antiopa* | 1 | 0 | 0.33 | 0.33 | 0.33 | 1.41 | 0.25 | 0.25 | 0.25 | 0.25 | 0.00 | 1 | 4 | 0.00 | 1.00 | 1 | 0.50 | 4 | hostplant | 5 | HOL | 32.0 |
| *Nymphalis polychloros* | 1 | 0 | 0.50 | 0.50 | 0.00 | 3.14 | 0.25 | 0.25 | 0.25 | 0.25 | 0.00 | 1 | 4 | 0.00 | 1.00 | 1 | 0.00 | 4 | hostplant | 4 | EUS | 28.5 |
| *Pararge aegeria* | 0 | 0 | 0.50 | 0.50 | 0.00 | 2.45 | 0.25 | 0.25 | 0.25 | 0.25 | 0.00 | 2 | 2 | 0.00 | 1.00 | 0 | 0.00 | 3 | leaf | 3 | EUR | 20.5 |
| *Polygonia c-album* | 0 | 0 | 0.50 | 0.50 | 0.00 | 3.09 | 0.20 | 0.20 | 0.20 | 0.20 | 0.20 | 2 | 3 | 0.00 | 1.00 | 0 | 0.50 | 4 | leaf | 4 | EUS | 23.0 |
| *Polygonia egea* | 0 | 0 | 0.50 | 0.50 | 0.00 | 1.00 | 0.25 | 0.25 | 0.25 | 0.25 | 0.00 | 2 | 3 | 0.00 | 1.00 | 0 | 0.00 | 4 | leaf | 4 | MED | 22.5 |
| *Pseudochazara anthelea* | 0 | 0 | 0.00 | 1.00 | 0.00 | 1.41 | 0.00 | 1.00 | 0.00 | 0.00 | 0.00 | 1 | 2 | 0.00 | 1.00 | 0 | 0.50 | 2 | out | 2 | MED | 24.0 |
| *Pyronia cecilia* | 0 | 0 | 0.33 | 0.33 | 0.33 | 1.00 | 0.00 | 0.00 | 1.00 | 0.00 | 0.00 | 1 | 2 | 0.00 | 1.00 | 0 | 0.00 | 2 | leaf | 2 | MED | 15.5 |
| **Scientific name** | ***Gregariousness*** | ***Myrmecophily*** | ***AL1*** | ***AL2*** | ***AL3*** | ***Feeding index*** | ***FL1*** | ***FL2*** | ***FL3*** | ***FL4*** | ***FL5*** | ***Generation numbers*** | ***Host plant apparency*** | ***LFM – flower*** | ***LFM – leaf*** | ***Migration*** | ***Mountain distribution*** | ***Overwintering stage*** | ***Ovum placement*** | ***Range size*** | ***Range type*** | ***Wingspan*** |
| *Pyronia tithonus* | 0 | 0 | 0.50 | 0.50 | 0.00 | 2.83 | 0.00 | 0.00 | 1.00 | 0.00 | 0.00 | 1 | 2 | 0.00 | 1.00 | 0 | 0.50 | 2 | out | 2 | EUR | 18.0 |
| *Satyrus ferula* | 0 | 0 | 0.50 | 0.50 | 0.00 | 1.00 | 0.00 | 0.00 | 1.00 | 0.00 | 0.00 | 1 | 2 | 0.00 | 1.00 | 0 | 0.50 | 2 | out | 4 | EUS | 27.5 |
| *Vanessa atalanta* | 0 | 0 | 0.33 | 0.33 | 0.33 | 1.41 | 0.20 | 0.20 | 0.20 | 0.20 | 0.20 | 1 | 2 | 0.00 | 1.00 | 1 | 0.50 | 4 | leaf | 5 | GLOB | 30.0 |
| *Vanessa cardui* | 0 | 0 | 0.33 | 0.33 | 0.33 | 5.73 | 0.20 | 0.20 | 0.20 | 0.20 | 0.20 | 3 | 1 | 0.00 | 1.00 | 1 | 0.50 | 4 | leaf | 5 | GLOB | 28.0 |

Information were compiled from existing literature (predominantly Tolman and Lewington [2], Bink [3], Garcia-Barros et al. [4], Hesselbarth et al. [5], Higgins and Riley [6], Lafranchis [7], Maravalhas [1], ProNatura [8], Tshikolovets [9]). Information specific for East Mediterranean (e. g., altitudinal range) were obtained from Pamperis [10]. Furthermore, information about overwintering stages and host plants originated from several faunistic works [11-20]. We used also existing trait datasets [21, 22].

**References**

1. De Jong YSDM. Fauna Europaea version 2.6. 2013 [cited 2014 7 July]. Available from: <http://www.faunaeur.org>.

2. Tolman T, Lewington R. Collins Butterfly Guide: The Most Complete Guide To The Butterflies Of Britain And Europe. London: HarperCollins; 2009.

3. Dobson M. Mammal distributions in the western Mediterranean: the role of human intervention. Mammal Review. 1998;28:77-88. doi: 10.1046/j.1365-2907.1998.00027.x.

4. Knop E, Kleijn D, Herzog F, Schmid B. Effectiveness of the Swiss agri-environment scheme in promoting biodiversity. Journal of Applied Ecology. 2006;43:120-7. doi: 10.1111/j.1365-2664.2005.01113.x.

5. Hesselbarth G, Van Oorschot H, Wagener S. Die Tagfalter der Türkei 1. Bocholt: Selbstverlag Sigbert Wagener; 1995.

6. Garcia-Barros E, Munguira ML, Stefanescu C, Moreno AV. Fauna Iberica. Madrid: Consejo Superior de Investigaciones Científicas; 2013.

7. Bunce RGH, Bell M, Farino T. The environmentally sensitive area legislation in the United Kingdom and its potential application to the Picos de Europa mountains in north-west Spain. Environmental Conservation. 1998;25:219-27. doi: 10.1017/s0376892998000277.

8. Van der Leeuw S. Vegetation Dynamics and Land Use in Epirus. Recent Dynamics of the Mediterranean Vegetation and Landscape: John Wiley & Sons, Ltd; 2004. p. 121-41.

9. Bink FA. Ecologische Atlas van de Dagvlinders van Noordwest-Europa. Haarlem: Schuyt & Co; 1992.

10. Pamperis LN. The butterflies of Greece. Athens: Bastas-Plessas Graphic Arts S.A.; 1997.

11. Heikkila M, Kaila L, Mutanen M, Pena C, Wahlberg N. Cretaceous origin and repeated tertiary diversification of the redefined butterflies. Proceedings of the Royal Society B-Biological Sciences. 2012;279:1093-9. doi: 10.1098/rspb.2011.1430.

12. Bishop TR, Botham MS, Fox R, Leather SR, Chapman DS, Oliver TH. The utility of distribution data in predicting phenology. Methods Ecol Evol. 2013;4:1024-32. doi: 10.1111/2041-210x.12112.

13. Van Swaay CAM, Nowicki P, Settele J, Van Strien AJ. Butterfly monitoring in Europe: methods, applications and perspectives. Biodivers Conserv. 2008;17:3455-69. doi: 10.1007/s10531-008-9491-4.

14. Vanderplank S, Ezcurra E, Delgadillo J, Felger R, McDade LA. Conservation challenges in a threatened hotspot: agriculture and plant biodiversity losses in Baja California, Mexico. Biodivers Conserv. 2014;23:2173-82. doi: 10.1007/s10531-014-0711-9.

15. Lopez-Villalta JS. Ecological trends in endemic Mediterranean butterflies. Bull Insectology. 2010;63:161-70.

16. Pro Natura. Les papillons de jour et leurs biotopes: espèces, dangers qui les menacent, protection. . Bâle: Ligue suisse pour la protection de la nature; 1987.

17. Konvicka M, Kuras T. Population structure, behaviour and selection of oviposition sites of an endangered butterfly, Parnassius mnemosyne, in Litovelske Pomoravi, Czech Republic. Journal of Insect Conservation. 1999;3:211-23. doi: 10.1023/a:1009641618795.

18. Kawahara AY, Breinholt JW. Phylogenomics provides strong evidence for relationships of butterflies and moths. Proceedings of the Royal Society B-Biological Sciences. 2014;281. doi: 20140970

10.1098/rspb.2014.0970.

19. Wiemers M. Hipparchia wyssii (Christ, 1889) Komplex: Beitrag zur Morphologie, Biologie, Ökologie und Verbreitung auf den Kanarischen Inseln (Lepidoptera, Satyridae). Nota lepid. 1991;14:255-78.

20. Garcia-Barros E. Delyed ovarian maturation in the butterfly *Hipparchia-semele* as a possible response to summer drought. Ecol Entomol. 1988;13:391-8. doi: 10.1111/j.1365-2311.1988.tb00371.x.

21. Cizek L, Fric Z, Konvicka M. Host plant defences and voltinism in European butterflies. Ecol Entomol. 2006;31:337-44. doi: 10.1111/j.1365-2311.2006.00783.x.

22. Garcia-Barros E, Romo H. The relationship between geographic range size and life history traits: is biogeographic history uncovered? A test using the Iberian butterflies. Ecography. 2010;33:392-401. doi: 10.1111/j.1600-0587.2010.06269.x.
