# Supplementary material for: Life History Traits Reflect Changes in Mediterranean Butterfly Communities Due to Forest Encroachment
Source: PLoS One. 2016 Mar 21;11(3):e0152026. doi: 10.1371/journal.pone.0152026 (PMC4801352; doi:10.1371/journal.pone.0152026)
Supplement: S1 Text — (DOCX) [file pone.0152026.s007.docx]

**Life History Traits Reflect Changes in Mediterranean Butterfly Communities due to Forest Encroachment**

**Short title: Forest Encroachment and Mediterranean Butterflies**

Jana Slancarova^1,2*^, Alena Bartonova^1,2^, Michal Zapletal^1,2^, Milan Kotilinek^1^, Zdenek Faltynek Fric^2^, Nikola Micevski^3^, Vasiliki Kati^4^, Martin Konvicka^1,2*^

^1^ Faculty of Science, University of South Bohemia, Ceske Budejovice, Czech Republic

^2^ Institute of Entomology, Biology Centre CAS, Ceske Budejovice, Czech Republic

^3^ Macedonian Entomological Society (ENTOMAK), Skopje, Republic of Macedonia (FYROM)

^4^ Department of Environmental and Natural Resources Management, University of Patras,

Agrinio, Greece

^*^ corresponding authors, emails: konva333@gmail.com (MK), slancaro@mail.com (JS)

**S1 Text.** **Detailed description of study regions, south Balkan, 2013–2014.**

**Study region R1**: Foothills of the Paramythia Mountains, north-western Greece (Epirus). Altitude of the sites 10–1000 m above sea level (mean: 365±288.7 SE), climate typically Mediterranean (average annual temperature 6–25 °C, average annual rainfall about 890 mm). Vegetation in lowland is formed by the Mediterranean maquis with *Quercus cocifera*, *Quercus ilex* and *Arbutus unedo*. Forest on the lower mountain slopes dominated by deciduous oaks, above 700 m a.s.l. it turns into mixed forest with *Fagus sylvatica*, *Tilia tomentosa*, *Abies borissiiregis*, *Pinus nigra* and *Pinus heldereichii*. Substrate mainly calcareous, in the northern part of Paramythia Mts. combined with flysch sediments. The lowland sites were situated partly on largely overgrown sea-facing slopes of the Ionian coast, partly on grazed or freshly abandoned grasslands above the Acheron River plains. The upland slopes were mainly at the eastern slopes of the mountains, amongst mostly depopulated villages near Vereniki village.

**Study region R2**: Foothills of Taygetos Mts., vicinity of Sparti, Peloponissos, Greece. Elevation range 50–1100 m a.s.l. (400±310.5), climate more temperate with oro-Mediterranean conditions (8–26 °C, 600 mm). Lowland is formed by a patchwork of arable land, Mediterranean maquis shrublands with *Quercus coccifera*, *Olea oleaster*, *Arbutus unedo* or *Pistacea lantiscus,* and coniferous forest with *Pinus pinea* and *P. halepensis*. Vegetation of mountain slopes is essentially made up of two conifer species: *Abies cephalonica* and *Pinus nigra*. Substrate is formed by limestones, dolomites and other calcareous sediments. Upland sites were situated at the eastern slopes of Taygetos Mts., above the settlement Mystras, most of the lowland sites were in the Eurotas River valley.

**Study region R3**: Northern coast of the Gulf of Corinth and southern slopes of Ghiona Mts. (Sterea Ellada, Greece). Altitude of sites varies between 200–800 m a.s.l. (531±183.9), is Mediterranean and much drier than in R1 and R2 (5–24 °C, 330 mm). Substrate is calcareous, mostly formed by carbonate-rich breccias. Vegetation is characterized by long-term human impacts and is a mostly formed by low maquis shrublands with *Quercus coccifera* and *Juniperus comunis* and other thorny scrubs, or by olive groves. In higher altitudes, *Abies cephalonica* prevails in evergreen forests. Upland sites were at the foothills of western slopes of the Ghiona Mts. (wider environs of Maladrino and Lidoriki villages), whereas lowland sites were mostly at south-oriented sea-facing slopes, between Erateini and Antikyra settlements.

**Study region R4**: Prilep environs (Macedonia). Altitude ranges from 200–1100 m a.s.l. (550±259.8), continental climate characterized by cold and humid winters and warm and dry summers (annual temperature 11–12 °C, 580 mm). Geology is variable and includes schist, gneiss and limestones. Lowland vegetation is dominated by mosaic of stony steppe with thorny bush – dominant shrub is *Paliurus spina-christi*, higher altitudes are mostly dry calcareous grasslands on shallow soils or broad-leaved deciduous forest (*Querco-Ostryetum carpinifolia*). The lowland sites were located within a largely cultivated area in the neighbourhood of Kavadartsi, upland sites were located in an apparently recently depopulated submontane area near Belovodica and Dunje villages.

**Study region R5**: Kardzhali environs (Eastern Rhodopes Mts., SE Bulgaria). Altitude between 200–600 m a.s.l. (364±126.8), climate transitional between continental and Mediterranean (2.5–25.1 °C, 760 mm). The woodlands consist mainly of deciduous oaks (*Quercus pubescens*, with a high proportion of *Pinus nigra*). and by thorny bush with *Paliorus spina-christi* or *Juniperus sp*. The lowland sites were partly in a cultivated area near Harmanli and partly in a former military area near Dinevo village, upland sites were situated near Vrelo village.
