# Supplementary material for: Life History Traits Reflect Changes in Mediterranean Butterfly Communities Due to Forest Encroachment
Source: PLoS One. 2016 Mar 21;11(3):e0152026. doi: 10.1371/journal.pone.0152026 (PMC4801352; doi:10.1371/journal.pone.0152026)
Supplement: S2 Text — (DOCX) [file pone.0152026.s008.docx]

**Life History Traits Reflect Changes in Mediterranean Butterfly Communities due to Forest Encroachment**

**Short title: Forest Encroachment and Mediterranean Butterflies**

Jana Slancarova^1,2*^, Alena Bartonova^1,2^, Michal Zapletal^1,2^, Milan Kotilinek^1^, Zdenek Faltynek Fric^2^, Nikola Micevski^3^, Vasiliki Kati^4^, Martin Konvicka^1,2*^

^1^ Faculty of Science, University of South Bohemia, Ceske Budejovice, Czech Republic

^2^ Institute of Entomology, Biology Centre CAS, Ceske Budejovice, Czech Republic

^3^ Macedonian Entomological Society (ENTOMAK), Skopje, Republic of Macedonia (FYROM)

^4^ Department of Environmental and Natural Resources Management, University of Patras,

Agrinio, Greece

^*^ corresponding authors, emails: konva333@gmail.com (MK), slancaro@mail.com (JS)

**S2 Text. Reference list to the sources of phylogenetic information.** We used a supertree built from the available phylogenetic information; formal genera and subgenera were adopted as nodes even in the absence of phylogenetic support. The sources were: Hesperiidae – Warren et al. [1]; Papilionidae – Aubert et al. [2], Caterino et al. [3]; Pieridae –Hesselbarth et al. [4], Pollock et al. [5], Brunton [6], Braby et al. [7], Back et al. [8]; Lycaenidae – Eliot [9], Fric et al. [10], Kandul et al. [11], Pech et al. [12], Talavera et al. [13], Vila et al. [14]; and Nymphalidae – Albre et al. , Aubert et al. [16], Martin et al. [17, 18], Kodandaramaiah & Wahlberg [19], Leneveu et al. [20], Nylin et al. [21], Nylin & Wahlberg [22], Pena et al. [24], Simonsen et al. [25, 26], Wahlberg [27], Wahlberg & Nylin [28], Wahlberg et al. [29-31]. Dennis et al. [32], Dinca et al. [33], Heikkilä et al. [34] and Wahlberg et al. [35] were adopted for the higher level taxonomy.

**References**

1. Wagner W. The genus Pyrgus in central Europe and its ecology - larval habitats, host plants and life cycles. Die Gattung Pyrgus in Mitteluropa und ihre Oekologie - Larvalhabitate, Naehrpflanzen und entwicklungszyklen. Abhandlungen aus dem Westfaelischen Museum fuer Naturkunde. 2006;68:83-122.

2. Warren AD, Ogawa JR, Brower AVZ. Revised classification of the family Hesperiidae (Lepidoptera: Hesperioidea) based on combined molecular and morphological data. Syst Entomol. 2009;34:467-523. doi: 10.1111/j.1365-3113.2008.00463.x.

3. Aubert J, Legal L, Descimon H, Michel F. Molecular phylogeny of swallowtail butterflies of the tribe Papilionini (Papilionidae, Lepidoptera). Mol Phylogenet Evol. 1999;12:156-67. doi: 10.1006/mpev.1998.0605.

4. Hesselbarth G, Van Oorschot H, Wagener S. Die Tagfalter der Türkei 1. Bocholt: Selbstverlag Sigbert Wagener; 1995.

5. Caterino MS, Reed RD, Kuo MM, Sperling FAH. A partitioned likelihood analysis of swallowtail butterfly phylogeny (Lepidoptera : papilionidae). Syst Biol. 2001;50:106-27. doi: 10.1080/106351501750107530.

6. Pollock DD, Watt WB, Rashbrook VK, Iyengar EV. Molecular phylogeny for Colias butterflies and their relatives (Lepidoptera : Pieridae). Ann Entomol Soc Am. 1998;91:524-31. doi: 10.1093/aesa/91.5.524

7. Brunton CFA. The evolution of ultraviolet patterns in European Colias butterflies (Lepidoptera, Pieridae): a phylogeny using mitochondrial DNA. Heredity. 1998;80:611-6.

8. Braby MF, Vila R, Pierce NE. Molecular phylogeny and systematics of the Pieridae (Lepidoptera : Papilionoidea): higher classification and biogeography. Zool J Linn Soc. 2006;147:238-75.

9. Back W, Miller MA, Opler PA. Genetic, Phenetic, and Distributional Relationships of Nearctic Euchloe (Pieridae, Pierinae, Anthocharidini). J Lepid Soc. 2011;65:1-14.

10. Eliot JN. Higher classification of the Lycaenidae (Lepidoptera): a tentative arrangement. Bull Br Mus Nat Hist Entomol. 1973;28:373-505.

11. Fric Z, Wahlberg N, Pech P, Zrzavy J. Phylogeny and classification of the Phengaris-Maculinea clade (Lepidoptera : Lycaenidae): total evidence and phylogenetic species concepts. Syst Entomol. 2007;32:558-67. doi: 10.1111/j.1365-3113.2006.00387.x.

12. Kandul NP, Lukhtanov VA, Dantchenko AV, Coleman JWS, Sekercioglu CH, Haig D, et al. Phylogeny of Agrodiaetus Hubner 1822 (Lepidoptera : Lycaenidae) inferred from mtDNA sequences of COI and COII and nuclear sequences of EF1-alpha: Karyotype diversification and species radiation. Syst Biol. 2004;53:278-98. doi: 10.1080/10635150490423692.

13. Pech P, Fric Z, Konvicka M, Zrzavy J. Phylogeny of Maculinea blues (Lepidoptera : Lycaenidae) based on morphological and ecological characters: evolution of parasitic myrmecophily. Cladistics-Int J Willi Hennig Soc. 2004;20:362-75. doi: 10.1111/j.1096-0031.2004.00031.x.

14. Tennent WJ. On the distribution of *Carcharodus tripolina* Verity, 1925, in north-west Africa (Lepioptera: Hesperiidae). Entomologist's Gazette. 1996;47:9-11.

15. Vila R, Lukhtanov VA, Talavera G, Gil F, Pierce NE. How common are dot-like distributions? Taxonomical oversplitting in western European Agrodiaetus (Lepidoptera: Lycaenidae) revealed by chromosomal and molecular markers. Biol J Linnean Soc. 2010;101:130-54. doi: 10.1111/j.1095-8312.2010.01481.x.

16. Jutzeler D, Lafranchis T, Verovnik R, Volpe G. Confirmation du rang spécifique d'Hipparchia syriaca Staudinger (1871) par élevage et examen en Gréce du principe d'exclusion d'H. fagi et syriaca que Lorković (1976) avait découvert en Dalmatie (Lepidoptera: Nymphalidae, Satyrinae). Entomologica romanica. 2009;14:5-12.

17. Albre J, Gers C, Legal L. Molecular phylogeny of the Erebia tyndarus (Lepidoptera, Rhopalocera, Nymphalidae, Satyrinae) species group combining CoxII and ND5 mitochondrial genes: A case study of a recent radiation. Mol Phylogenet Evol. 2008;47:196-210. doi: 10.1016/j.ympev.2008.01.009.

18. Aubert J, Barascud B, Descimon H, Michel F. Molecular systematics of the Argynninae (Lepidoptera:Nymphalidae). Comptes Rendus Acad Sci Ser III-Sci Vie-Life Sci. 1996;319:647-51.

19. Martin JF, Gilles A, Descimon H. Molecular phylogeny and evolutionary patterns of the European satyrids (Lepidoptera : Satyridae) as revealed by mitochondrial gene sequences. Mol Phylogenet Evol. 2000;15:70-82. doi: 10.1006/mpev.2000.0757.

20. Martin JF, Gilles A, Lortscher M, Descimon H. Phylogenetics and differentiation among the western taxa of the Erebia tyndarus group (Lepidoptera : Nymphalidae). Biol J Linnean Soc. 2002;75:319-32.

21. Kodandaramaiah U, Wahlberg N. Phylogeny and biogeography of Coenonympha butterflies (Nymphalidae: Satyrinae) - patterns of colonization in the Holarctic. Syst Entomol. 2009;34:315-23. doi: 10.1111/j.1365-3113.2008.00453.x.

22. Leneveu J, Chichvarkhin A, Wahlberg N. Varying rates of diversification in the genus Melitaea (Lepidoptera: Nymphalidae) during the past 20 million years. Biol J Linnean Soc. 2009;97:346-61. doi: 10.1111/j.1095-8312.2009.01208.x.

23. Nylin S, Wahlberg N. Does plasticity drive speciation? Host-plant shifts and diversification in nymphaline butterflies (Lepidoptera : Nymphalidae) during the tertiary. Biol J Linnean Soc. 2008;94:115-30. doi: 10.1111/j.1095-8312.2008.00964.x.

24. Nylin S, Nyblom K, Ronquist F, Janz N, Belicek J, Kallersjo M. Phylogeny of Polygonia, Nymphalis and related butterflies (Lepidoptera : Nymphalidae): a total-evidence analysis. Zool J Linn Soc. 2001;132:441-68. doi: 10.1111/j.1096-3642.2001.tb02470.x.

25. Pena C, Wahlberg N, Weingartner E, Kodandaramaiah U, Nylin S, Freitas AVL, et al. Higher level phylogeny of Satyrinae butterflies (Lepidoptera : Nymphalidae) based on DNA sequence data. Mol Phylogenet Evol. 2006;40:29-49. doi: 10.1016/j.ympev.2006.02.007.

26. Pena C, Nylin S, Wahlberg N. The radiation of Satyrini butterflies (Nymphalidae: Satyrinae): a challenge for phylogenetic methods. Zool J Linn Soc. 2011;161:64-87. doi: 10.1111/j.1096-3642.2009.00627.x.

27. Simonsen TJ, Wahlberg N, Brower AVZ, de Jong R. Morphology, molecules and fritillaries: approaching a stable phylogeny for Argynnini (Lepidoptera : Nymphalidae). Insect Syst Evol. 2006;37:405-18. doi: 10.1163/187631206788831407.

28. Simonsen TJ, Wahlberg N, Warren AD, Sperling FAH. The evolutionary history of Boloria (Lepidoptera: Nymphalidae): phylogeny, zoogeography and larval-foodplant relationships. Syst Biodivers. 2010;8:513-29. doi: 10.1080/14772000.2010.532833.

29. Wahlberg N. The phylogenetics and biochemistry of host-plant specialization in Melitaeine butterflies (Lepidoptera : Nymphalidae). Evolution. 2001;55:522-37. doi: 10.1554/0014-3820(2001)055[0522:tpaboh]2.0.co;2.

30. Wahlberg N, Nylin S. Morphology versus molecules: resolution of the positions of Nymphalis, Polygonia, and related genera (Lepidoptera : Nymphalidae). Cladistics-Int J Willi Hennig Soc. 2003;19:213-23. doi: 10.1016/s0748-3007(03)00027-6.

31. Wahlberg N, Weingartner E, Nylin S. Towards a better understanding of the higher systematics of Nymphalidae (Lepidoptera : Papilionoidea). Mol Phylogenet Evol. 2003;28:473-84. doi: 10.1016/s1055-7903(03)00052-6.

32. Wahlberg N, Brower AVZ, Nylin S. Phylogenetic relationships and historical biogeography of tribes and genera in the subfamily Nymphalinae (Lepidoptera : Nymphalidae). Biol J Linnean Soc. 2005;86:227-51. doi: 10.1111/j.1095-8312.2005.00531.x.

33. Wahlberg N, Weingartner E, Warren AD, Nylin S. Timing major conflict between mitochondrial and nuclear genes in species relationships of Polygonia butterflies (Nymphalidae: Nymphalini). BMC Evol Biol. 2009;9. doi: 10.1186/1471-2148-9-92.

34. Dennis RLH, Donato B, Sparks TH, Pollard E. Ecological correlates of island incidence and geographical range among British butterflies. Biodivers Conserv. 2000;9:343-59. doi: 10.1023/a:1008924329854.

35. Dinca V, Zakharov EV, Hebert PDN, Vila R. Complete DNA barcode reference library for a country's butterfly fauna reveals high performance for temperate Europe. Proceedings of the Royal Society B-Biological
